# Establishment of a machine learning prediction model for Wallerian degeneration after ischemic stroke

**DOI:** 10.3389/fmed.2026.1841202

**Published:** 2026-06-30

**Authors:** Zhiqi Yu, Xuhui Liu, Xujie Wang, Siyue Chen, Wei Tang

**Affiliations:** 1 Department of Neurology, Xinhua Hospital Affiliated with Dalian University, Dalian, Liaoning, China; 2 Department of Neurology, The Second Hospital of Lanzhou University, Lanzhou, Gansu, China; 3 Department of Emergency ICU, The Affiliated Hospital of Qinghai University, Xining, Qinghai, China; 4 Department of Neurology, Fifth Affiliated Hospital of Xinjiang Medical University, Urumqi, Xinjiang, China

**Keywords:** ischemic stroke, machine learning, random forest, SHAP, Wallerian degeneration

## Abstract

**Background:**

Wallerian degeneration (WD) is a common and clinically significant complication of ischemic stroke (IS). Due to the multifactorial and nonlinear characteristics of its underlying mechanisms, accurately identifying high-risk patients early remains challenging. This study aimed to develop and validate an interpretable machine learning (ML) model to predict WD after IS.

**Methods:**

We retrospectively analyzed clinical data from 269 patients with IS, all admitted to the Xinhua Hospital of Dalian University. The patients were randomly divided into a training set (70%) and an internal validation set (30%). Thirty demographic, imaging, and laboratory variables were assessed, and predictive features were selected through Least Absolute Shrinkage and Selection Operator (LASSO) regression, followed by confirmation using multivariate logistic regression. Nine ML algorithms were constructed and compared. The best-performing model was interpreted using Shapley Additive Explanations (SHAP).

**Results:**

Among the 269 patients, 35.32% (95/269) of IS patients developed WD. LASSO regression selected eight candidate predictors (six reaching statistical significance in multivariate logistic regression; MCA and PCA retained based on LASSO selection and biological relevance): smoking history, hyperlipidemia, standard antiplatelet therapy, achieving LDL target with oral statins, maximum cross-sectional area of the stroke, middle cerebral artery (MCA), posterior cerebral artery (PCA), and the number of stroke-affected layers. Within the study population, the Random Forest model showed internally favorable predictive performance (training AUC = 0.946; validation AUC = 0.856) and reasonable internal consistency, surpassing AdaBoost, Logistic Regression, Lasso, Decision Tree, KNN, GaussianNB, XGBoost, and LightGBM. Through SHAP analysis, this study quantified and visualized the contribution of each predictive variable to the Random Forest model’s prediction of the occurrence of WD, identifying key factors such as smoking history, MCA, and PCA, and revealing their interactions, thereby enhancing the model’s interpretability for research purposes.

**Conclusion:**

We developed and validated an interpretable Random Forest model with potential for predicting the occurrence of WD. By integrating demographic, imaging, and laboratory features, this model provides an internally validated framework that shows promise for early risk assessment, with potential to support personalized management pending external validation.

## Introduction

Ischemic stroke (IS) is one of the leading causes of disability and death in adults and is recognized as a significant public health challenge (1). Among individuals aged ≥ 50 years, IS is the primary factor contributing to the loss of disability-adjusted life years (DALYs) (2, 3). Despite the presence of efficient stroke emergency systems (such as thrombolysis and thrombectomy), patients often face poor prognoses and experience sequelae such as hemiplegia, aphasia, and cognitive impairment, significantly affecting their quality of life (4). In the central nervous system, IS is the most common cause of Wallerian degeneration (WD) (5). WD is a degenerative process in which distal axons undergo anterograde or retrograde demyelination following injury to the neuron or proximal axon (6). WD patients may present with clinical manifestations including varying degrees of cognitive and psychiatric disorders, motor dysfunction, ataxia, and other symptoms (7, 8). WD occurring after IS directly impacts the long-term recovery of motor functions, prognosis, and quality of life (9). Therefore, developing a useful early risk assessment tool during follow-up can help capture the clinical diagnostic timing, enhance clinical monitoring of high-risk patients, and promote timely assessment of complications, thereby improving prognosis and quality of life for patients.

The pathophysiological mechanisms of WD involve a multifactorial process, including axonal degeneration, myelin rupture, coagulation dysfunction, inflammatory cascades, and more (10–12). Previous studies have identified several relevant risk factors, including smoking history, middle cerebral artery (MCA) involvement, and the time interval from symptom onset to MRI scanning (13, 14). In addition, comorbidities such as hyperlipidemia and atrial fibrillation, as well as laboratory indicators including PLT count and low-density lipoprotein levels, are also considered to be associated with WD (15–18). However, the predictive ability of traditional regression-based models remains limited. These models typically assume linear relationships between variables, making them difficult to apply to high-dimensional data, and are unable to fully capture the complex nonlinear interactions involved in the WD process.

In contrast, machine learning (ML) algorithms offer a promising alternative that can handle nonlinear relationships and extract underlying patterns from complex high-dimensional datasets (19). In recent years, ML has been successfully applied to prognostic prediction in various neurological and critical diseases, including spontaneous intracerebral hemorrhage, IS, sepsis, and traumatic brain injury (20, 21). However, research on WD occurring after IS remains relatively limited, and the results are heterogeneous. Existing prediction methods have not been fully validated and are difficult to apply directly in routine clinical follow-up practice. Many existing methods, especially those relying on linear assumptions or integrating only a limited number of features, often lack sufficient interpretability and have not yet yielded tools ready for clinical application pending external validation. For instance, a retrospective case series analysis explored Wallerian degeneration of the corticospinal tract following multimodal treatment of high-grade gliomas and its association with radiation dose exposure. Although the study provides valuable insights into identifying high-risk patients, it is limited by the absence of a formal predictive risk model, its retrospective design, small sample size, and lack of standardized functional assessments (22). Moreover, WD is a key endpoint with clear clinical intervention value (23): by applying clinical prediction models when patients experience recurrent IS, individualized WD risk assessments can be provided, helping with the timely assessment of complications, advancing treatment windows, delaying or even preventing disease progression, and improving patient prognosis. Given the limited and heterogeneous existing evidence (24, 25), as well as the difficulty in fully capturing the complex nonlinear relationships between high-dimensional clinical, laboratory, and imaging predictive indicators, we propose an interpretable modeling framework guided by clinical application and conduct a comprehensive evaluation (including discrimination, calibration, and decision analysis utility) to predict the occurrence of WD in patients with IS and assess its clinical value in early risk assessment to support clinical management decisions.

To address these gaps, we formulated three specific research objectives: (1) to systematically compare the predictive performance of nine commonly used ML algorithms within a unified feature set and evaluation framework; (2) to identify the best-performing model for predicting WD occurrence and validate its stability through comprehensive evaluation metrics including discrimination, calibration, and decision analysis; and (3) to enhance model interpretability through SHAP-based analysis, thereby providing a transparent, potentially applicable decision support framework for early WD risk assessment pending external validation.

Compared to traditional regression methods, ML can better handle the nonlinear interactions and complex joint effects between imaging, coagulation, inflammation, and metabolic indicators (26, 27). We compared nine commonly used machine learning algorithms within a unified feature set and evaluation framework (including discrimination, calibration, and decision analysis utility) and emphasized the clinical translational value of the model through SHAP-based interpretability analysis, providing a transparent framework for risk assessment. Specifically, the goal of this study is to compare the model performance of different algorithms, select the best-performing predictive model, and provide an interpretable tool for clinical assessment that translates model predictions into clinically meaningful risk assessments, thus translating model outputs into actionable clinical insights.

## Materials and methods

### Study design, data collection, and ethical considerations

**FIGURE 1:**
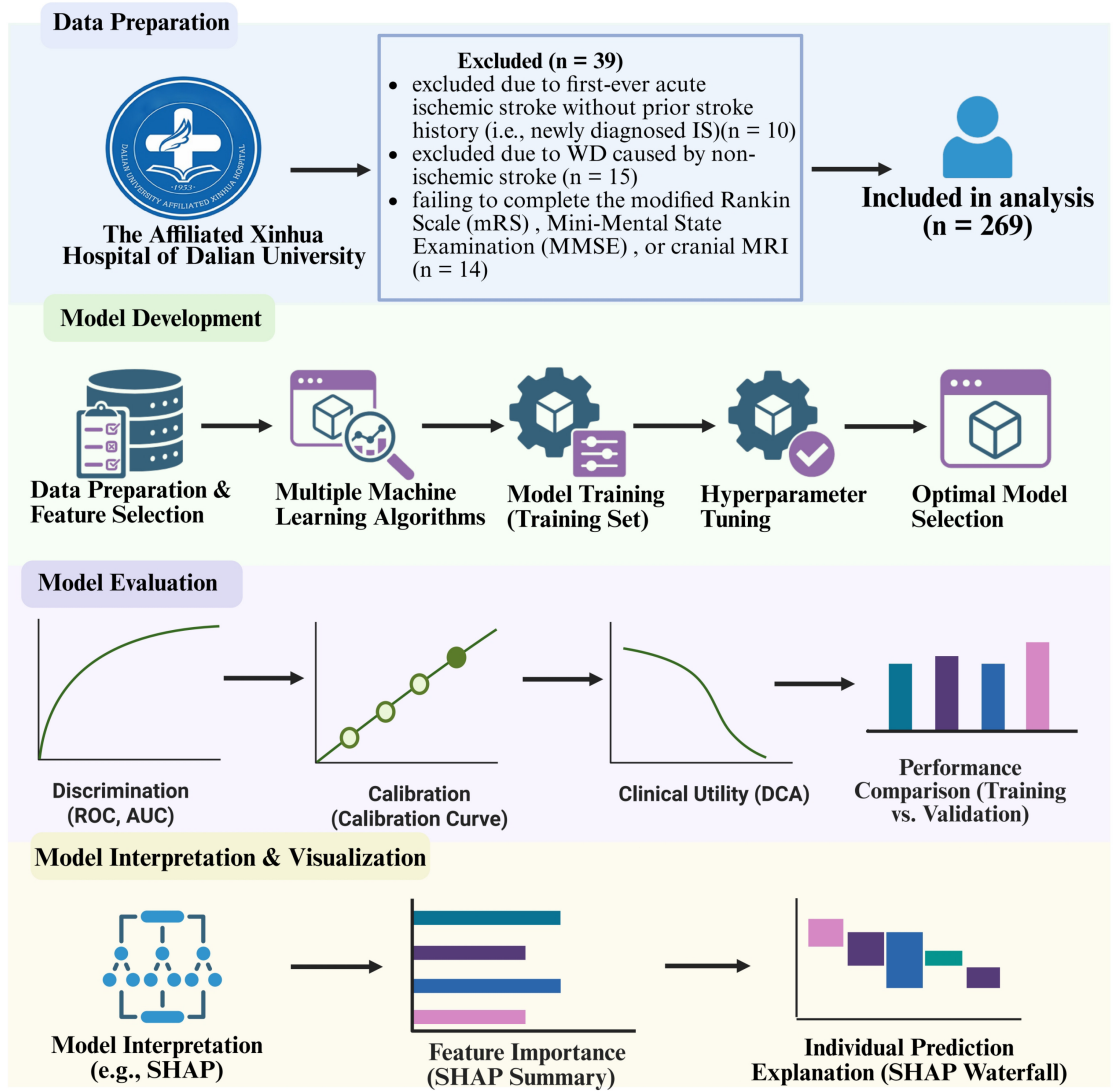
Flowchart of the study analysis. After excluding 39 cases from 308 based on prespecified criteria, 269 patients were included. The pipeline involved model development (nine algorithms, tuning, selection), evaluation (discrimination, calibration, clinical utility, comparison), and SHAP-based interpretation. A detailed summary of participant enrollment, inclusion, and exclusion numbers is provided in tabular format in Supplementary Table 1 and Supplementary Figure 1 for enhanced transparency and reproducibility. IS, ischemic stroke; WD, Wallerian degeneration; mRS, modified Rankin Scale; MMSE, Mini-Mental State Examination.

This retrospective observational study was conducted at Xinhua Hospital of Dalian University, following the RECORD (Reporting of Studies Conducted Using Observational Routinely-Collected Health Data) guidelines. Clinical and imaging data of IS patients admitted between January 2022 and June 2024 were extracted from the hospital’s electronic medical record system. After strict inclusion and exclusion criteria, a total of 269 patients were included in the study. During the study period, a total of 308 IS patient records were screened. Of these, 10 were excluded due to first-ever acute ischemic stroke without prior stroke history (i.e., newly diagnosed IS), 15 were excluded due to WD caused by non-ischemic stroke, and14 were excluded for failing to complete the modified Rankin Scale (mRS) (28), Mini-Mental State Examination (MMSE) (29), or cranial MRI. Ultimately, 269 patients were included in the analysis. The study protocol was reviewed and approved by the Ethics Committee of Xinhua Hospital of Dalian University (Ethics No.: 2024-12-01). As this is a retrospective analysis, the data used were anonymized and did not involve identifiable patient information, thus exempting the requirement for informed consent. All study procedures complied with institutional and national privacy and confidentiality standards. A summary flowchart of the study screening, exclusion, and cohort allocation is shown in Figure 1, Supplementary Figure 1 and Supplementary Table 1.

### Inclusion and exclusion criteria, cohort allocation

The study population included patients aged ≥ 18 years with a history of ≥ 1 previous IS who underwent cranial MRI assessment for WD. The inclusion criteria were: (1) age ≥ 18 years; (2) history of ≥ 1 previous IS; (3) availability of cranial MRI data adequate for WD assessment. The exclusion criteria were: (1) first-ever acute ischemic stroke without prior stroke history (newly diagnosed IS); (2) WD caused by non-ischemic stroke; (3) inability to complete the mRS scale, MMSE scale assessment, or cranial MRI due to other reasons; (4) long-term alcohol consumption, drug abuse, or use of medications affecting cognitive function, such as antidepressants, antipsychotics, sedatives, or hypnotics; (5) severe heart, lung, or renal dysfunction. After screening, a total of 269 patients were included and randomly assigned to the training cohort (*n* = 188) and the validation cohort (*n* = 81) at a 7:3 ratio for model development and internal validation. WD was the primary binary outcome variable, not an inclusion criterion. Patients were classified into the WD group (*n* = 95, 35.32%) and the non-WD group (*n* = 174, 64.68%) based on imaging findings of asymmetric mesencephalic cerebral peduncles. The 70:30 data split was performed prior to any feature selection or model development, with all analytical procedures conducted exclusively within the training set to prevent information leakage (Supplementary Figure 2).

### Study variables

The primary outcome of this study was the occurrence of WD after IS, defined as WD diagnosed on imaging with asymmetric mesencephalic cerebral peduncles. We selected 30 variables, including demographic characteristics (age, sex, hypertension, smoking, hyperlipidemia, diabetes, and atrial fibrillation), stroke-related treatment variables (standardized antiplatelet therapy, standardized oral antihypertensive treatment, standardized oral lipid-lowering treatment, and standardized neurological rehabilitation), clinical scale assessment [NIHSS motor score, derived from the National Institutes of Health Stroke Scale (30)], laboratory parameters [creatinine (Cr), blood urea nitrogen (BUN), red blood cell count (RBC), platelet count (PLT), white blood cell count (WBC), fibrinogen (FIB), D-dimer, and homocysteine (HCY)], and imaging features (the location, size, and responsible vessels of IS, as well as the number of stroke-affected layers).

### Statistical analysis methods

Univariate analyses were first conducted using χ^2^ or Fisher’s exact tests for categorical variables and Mann–Whitney U tests for continuous variables. Variables with *p* < 0.05 were further screened using the least absolute shrinkage and selection operator (LASSO) logistic regression with 10-fold cross-validation to minimize overfitting. The optimal parameter (λ = −2.9082) was selected based on the 1-SE rule, resulting in eight predictive variables with non-zero coefficients. These predictive variables were then entered into a multivariate logistic regression model with backward stepwise selection, and variables with non-zero LASSO coefficients were retained as candidate predictors based on statistical selection and biological plausibility. In addition, variance inflation factors (VIFs) were assessed to examine collinearity, and no significant multicollinearity was detected.

### Machine learning model development and performance evaluation

These eight independent predictive factors (smoking history, hyperlipidemia, standardized antiplatelet therapy, achieving LDL target with oral statins, maximum cross-sectional area of the stroke, MCA, posterior cerebral artery (PCA), and the number of stroke-affected layers) were used to construct nine ML models: Random Forest, AdaBoost, Logistic Regression, Lasso regression, DecisionTree, KNN, GaussianNB, XGBoost, and LightGBM. The performance of each model was comprehensively evaluated from three aspects: discrimination, calibration, and clinical applicability. Discrimination was quantified using the area under the receiver operating characteristic curve (AUC), precision-recall analysis, and metrics based on the confusion matrix [including sensitivity, specificity, precision, recall, F1 score, positive predictive value (PPV), and negative predictive value (NPV)]. Calibration was assessed using calibration plots, the Hosmer-Lemeshow goodness-of-fit test, and the Brier score (31). Clinical applicability was evaluated through decision curve analysis (DCA), and in subgroup analysis, the area under the precision-recall curve (AUPRC) was also used to address potential class imbalance issues.

### Model interpretability with SHAP

Among the nine models, the Random Forest model demonstrated internally favorable predictive performance with reasonable internal consistency within the study population. To enhance the interpretability of the model, the Shapley Additive Explanations (SHAP) method was used to quantify the contribution of each predictive factor to the model’s output. The SHAP summary plot showed that smoking history, MCA, and PCA contributed the most to the predictions, followed by standardized antiplatelet therapy, maximum cross-sectional area of the stroke, achieving LDL target with oral statins, the number of stroke-affected layers, and hyperlipidemia.

### Statistical comparisons and software

Categorical variables were presented as counts and percentages, and continuous variables were summarized as medians and interquartile ranges. Statistical comparisons between groups were conducted using the chi-square test (χ^2^) or Fisher’s exact test for categorical variables and the Mann-Whitney U test for continuous variables. The logistic regression model reported odds ratios (OR) and their 95% confidence intervals (CI). All statistical analyses were performed using R software (version 4.2.1) and Python (version 3.6.5), with a two-tailed *p*-value < 0.05 considered statistically significant. With 95 WD events and 8 predictors, the events-per-variable (EPV) ratio was 11.9, exceeding the recommended minimum of 10 (Supplementary Figure 3). Extended subgroup analyses (Supplementary Table 21), detailed confusion matrices (Supplementary Table 22), variable temporal classification (Supplementary Table 23), treatment adequacy analysis (Supplementary Table 24), and additional figures (Supplementary Figures 21–24) are provided. Bootstrap validation (500 iterations) yielded a median AUC of 0.859 (95% CI: 0.753–0.944) for Random Forest (Supplementary Figure 25 and Supplementary Table 25). This study was reported in accordance with the TRIPOD guidelines (Supplementary Table 26). No missing data were present in the final analytical dataset. Software: Python 3.8.10 with scikit-learn 1.2.2, R 4.2.1. Analysis code is available upon reasonable request.

## Results

A total of 269 patients with IS were included in this study, of which 175 were male (65.06%) and 94 were female (34.94%), with a median age of 70.00 years [interquartile range (IQR): 65.00–77.00 years]. Of these, 188 patients were randomly assigned to the training group and 81 to the validation group in a 7:3 ratio. The overall incidence of WD was 35.32% (*n* = 95). As shown in Supplementary Table 2, the training and validation groups were generally balanced in baseline demographic and clinical characteristics, with no significant differences in age, hypertension, diabetes, atrial fibrillation, imaging characteristics, or key laboratory indicators (all *p* > 0.05). In contrast, there were significant differences between the WD and non-WD groups, as shown in Table 1. Smoking was more common in the WD group (60% vs. 40%, *p* < 0.001). Hyperlipidemia was more prevalent in the WD group (64.21% vs. 35.79%, *p* < 0.001). Imaging characteristics such as the maximum cross-sectional area of the stroke (56.84% vs. 43.16%) and responsible blood vessels being the MCA (66.32% vs. 33.68%) were higher in the WD group, with both *p* < 0.001, and the responsible blood vessel being PCA was also more frequent (13.68% vs. 86.32%, *p* < 0.001). Laboratory results showed significant differences between the two groups in whether the standardized antiplatelet therapy targets were met. The WD group had a higher incidence of failing to meet the antiplatelet therapy targets (40.00% vs. 60.00%, *p* < 0.001). The factors with no significant differences in baseline clinical and laboratory characteristics between the WD and non-WD groups are shown in Supplementary Table 3.

**TABLE 1:** Comparison of baseline clinical and laboratory characteristics between IS patients with and without WD showed statistically significant differences.

Variables	Total (*N* = 269)	No (*N* = 174)	Yes (*N* = 95)	*P*-value
Smoke, *n* (%)				< 0.001
No	191 (71.00%)	153 (87.93%)	38 (40.00%)	
Yes	78 (29.00%)	21 (12.07%)	57 (60.00%)	
Hyperlipidemia, *n* (%)				< 0.001
No	149 (55.39%)	115 (66.09%)	34 (35.79%)	
Yes	120 (44.61%)	59 (33.91%)	61 (64.21%)	
MCSA, *n* (%)				< 0.001
No	158 (58.74%)	117 (67.24%)	41 (43.16%)	
Yes	111 (41.26%)	57 (32.76%)	54 (56.84%)	
MCA, *n* (%)				< 0.001
No	145 (53.90%)	113 (64.94%)	32 (33.68%)	
Yes	124 (46.10%)	61 (35.06%)	63 (66.32%)	
PCA, *n* (%)				< 0.001
No	183 (68.03%)	101 (58.05%)	82 (86.32%)	
Yes	86 (31.97%)	73 (41.95%)	13 (13.68%)	
APT Standard, *n* (%)				< 0.001
No	119 (44.24%)	62 (35.63%)	57 (60.00%)	
Yes	150 (55.76%)	112 (64.37%)	38 (40.00%)	

MCSA, Maximum Cross-Sectional Area; MCA, Middle Cerebral Artery; PCA, Posterior Cerebral Artery; APT Standard, Antiplatelet Therapy-Standard.

### Variable selection through LASSO regression

**FIGURE 2:**
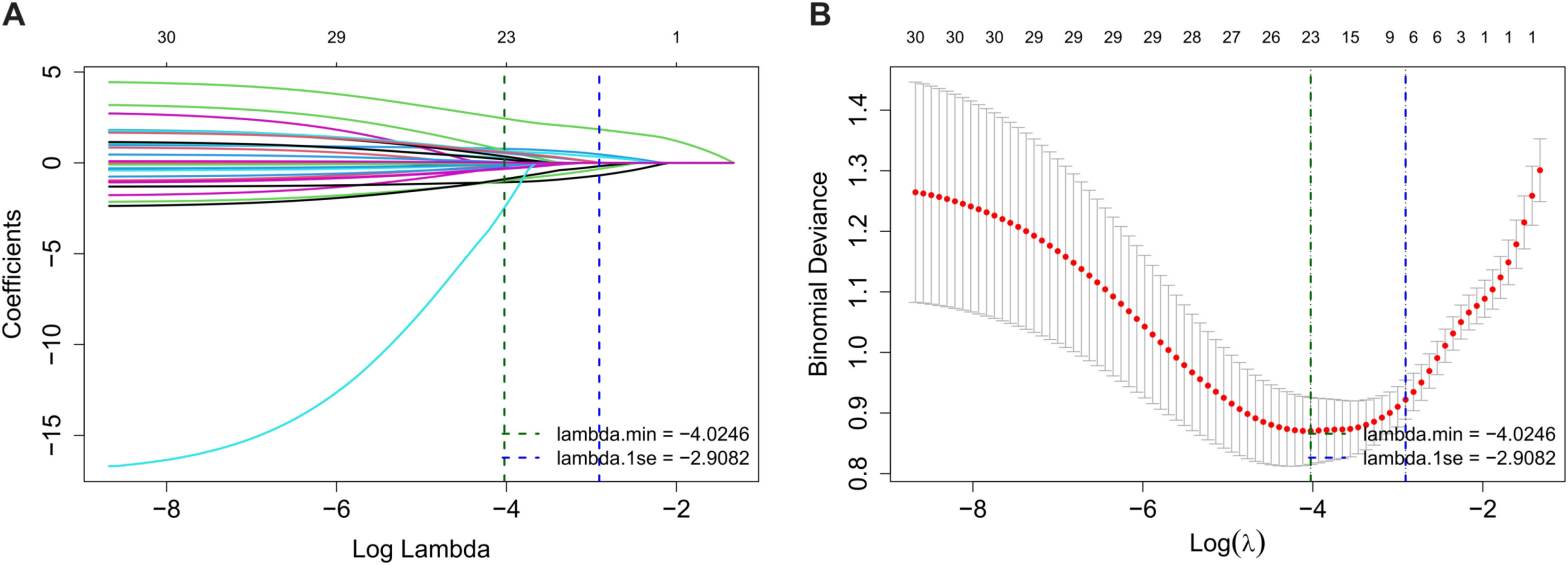
Feature selection using least absolute shrinkage and selection operator (LASSO) logistic regression. **(A)** Coefficient profile plot showing the trajectories of 30 candidate variable coefficients as a function of the regularization parameter log(λ). As log(λ) increases (stronger regularization), coefficients progressively shrink toward zero, with only the most predictive variables retaining non-zero values. Each colored line represents one variable. **(B)** Ten-fold cross-validation curve showing binomial deviance (*y*-axis) as a function of log(λ) (*x*-axis). The left vertical dashed line indicates the λ value yielding minimum deviance (λ.min), and the right vertical dashed line indicates the λ value at one standard error above the minimum (λ.1se = - 2.9082), which was selected as the optimal regularization level to enhance model generalizability. At this optimal level, eight predictive variables with non-zero coefficients were retained. Error bars represent ± 1 standard error from 10-fold cross-validation. LASSO, Least Absolute Shrinkage and Selection Operator.

To identify the most predictive variables and avoid overfitting, we employed the Least Absolute Shrinkage and Selection Operator (LASSO) logistic regression model and used 10-fold cross-validation. The selection of the parameter λ was based on the minimum standard and the 1-standard error (1-SE) rule. As shown in Figure 2A, with increasing log(λ), the regression coefficients of most variables gradually shrank toward zero, illustrating the variable selection process of the LASSO regression. Figure 2B presents the cross-validated binomial deviance curve in the primary analysis cohort, with the vertical dashed lines indicating the λ value corresponding to the minimum deviance (Lambda.min) and that corresponding to the 1-SE rule (Lambda.1se), respectively. To enhance the model’s generalizability, we adopted the 1-SE criterion and selected Lambda.1se = −2.9082 as the optimal point. In the extended analysis (see Supplementary Figure 4), a consistent pattern of minimized bias across folds was observed, further validating the stability of our model selection process. At this optimal level, the final model retained eight predictive factors with non-zero coefficients (see Supplementary Table 4): smoking history (β = 1.84478059), hyperlipidemia (β = 0.46858223), standardized antiplatelet therapy (β = −0.68976836), achieving LDL target with oral statins (β = −0.33651309), maximum cross-sectional area of the stroke (β = 0.0127935), MCA (β = 0.33613374), PCA (β = −0.18175487), and the number of stroke-affected layers (β = 0.01813518).

### Multivariate logistic regression identifies independent predictors of WD occurrence

**FIGURE 3:**
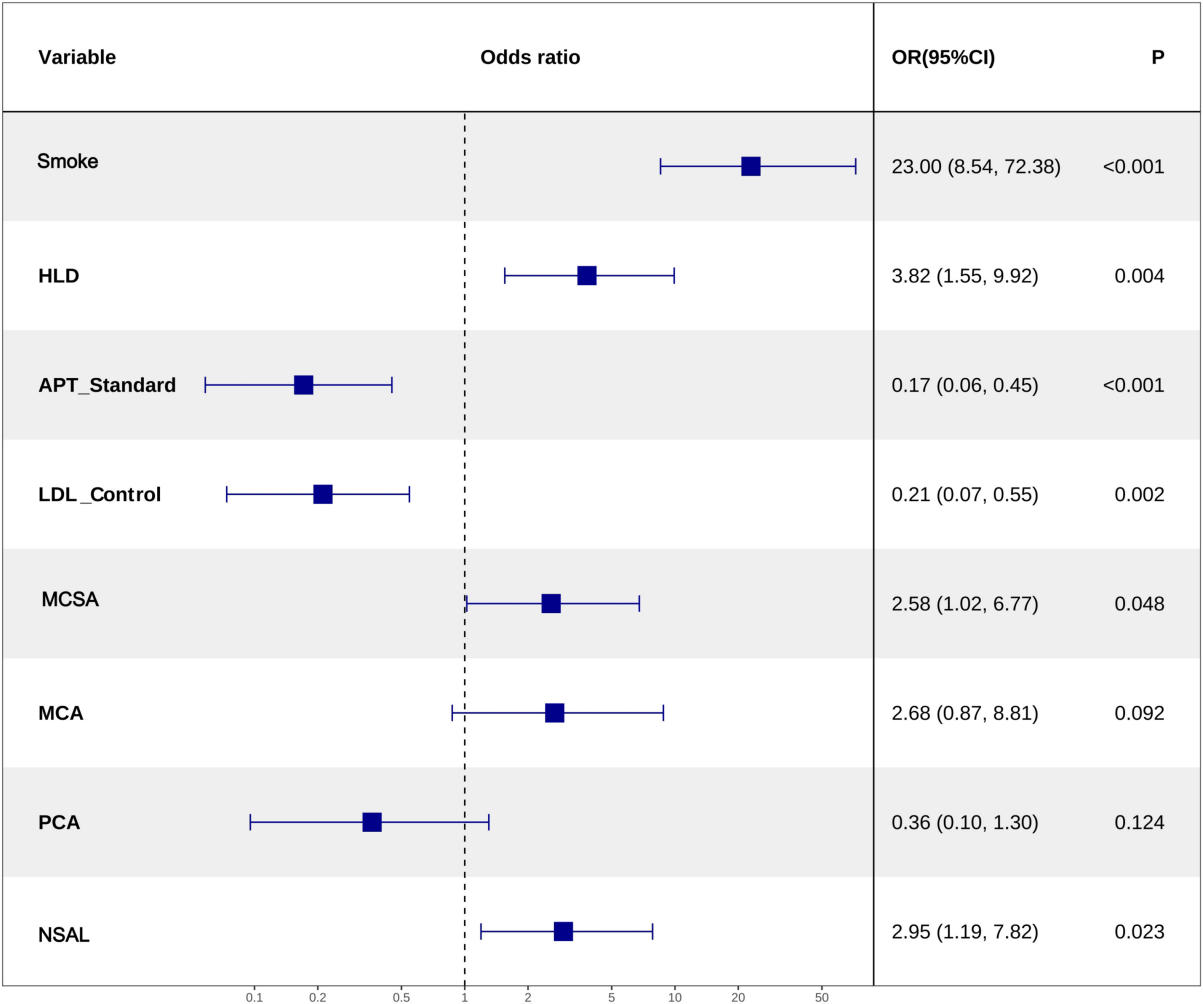
Forest plot of multivariate logistic regression analysis identifying independent predictors of Wallerian degeneration (WD). The *x*-axis represents odds ratios (ORs) on a logarithmic scale, with the vertical dashed line at OR = 1.0 indicating no effect. Each horizontal bar represents the 95% confidence interval (CI) for a predictor. Red circles mark the point estimates. Variables with ORs significantly above 1.0 are risk factors (e.g., smoking history: OR = 23, 95% CI: 8.54–72.38, *p* < 0.001), while variables with ORs below 1.0 are protective factors (e.g., standardized antiplatelet therapy: OR = 0.17, 95% CI: 0.06–0.45, *p* < 0.001). MCA (OR = 2.68, *p* = 0.092) and PCA (OR = 0.36, *p* = 0.124) showed trends toward association but did not reach conventional significance; they were retained based on LASSO selection and biological plausibility. OR, odds ratio; CI, confidence interval; MCA, middle cerebral artery; PCA, posterior cerebral artery; APT, antiplatelet therapy; LDL-C, low-density lipoprotein cholesterol.

To further identify the independent predictors of WD occurrence, all variables selected by LASSO regression were entered into a multivariate logistic regression model and analyzed using backward stepwise selection. As shown in Figure 3, eight variables were significantly associated with the outcome. Smoking history was independently associated with an increased risk of WD (OR = 22.999, 95% CI: 7.981–66.278, *p* < 0.001). Patients with hyperlipidemia had a higher likelihood of WD progression compared to those without hyperlipidemia (OR = 3.816, 95% CI: 1.520–9.581, *p* < 0.01). MCA (OR = 2.68, 95% CI: 0.851–8.441, *p* = 0.092) and PCA (OR = 0.362, 95% CI: 0.099–1.323, *p* = 0.124) showed trends toward association with WD but did not reach conventional statistical significance (*p* < 0.05) in multivariate logistic regression. Both variables were retained based on LASSO selection (non-zero coefficients) and their established biological relevance. The maximum cross-sectional area of the stroke (OR = 2.577, 95% CI: 1.010–6.577, *p* < 0.05) and the number of stroke-affected layers (OR = 2.95, 95% CI: 1.163–7.487, *p* < 0.05) were also identified as risk factors for WD. Irregular antiplatelet therapy (OR = 0.171, 95% CI: 0.062–0.471, *p* < 0.001) and achieving LDL-C control targets with oral statins (OR = 0.212, 95% CI: 0.079–0.571, *p* < 0.01) was identified as a protective factor against WD. To verify the collinearity of core variables, multicollinearity was excluded using the variance inflation factor (VIF), with specific data provided in Supplementary Table 5.

### Machine-learning model development and evaluation

**FIGURE 4:**
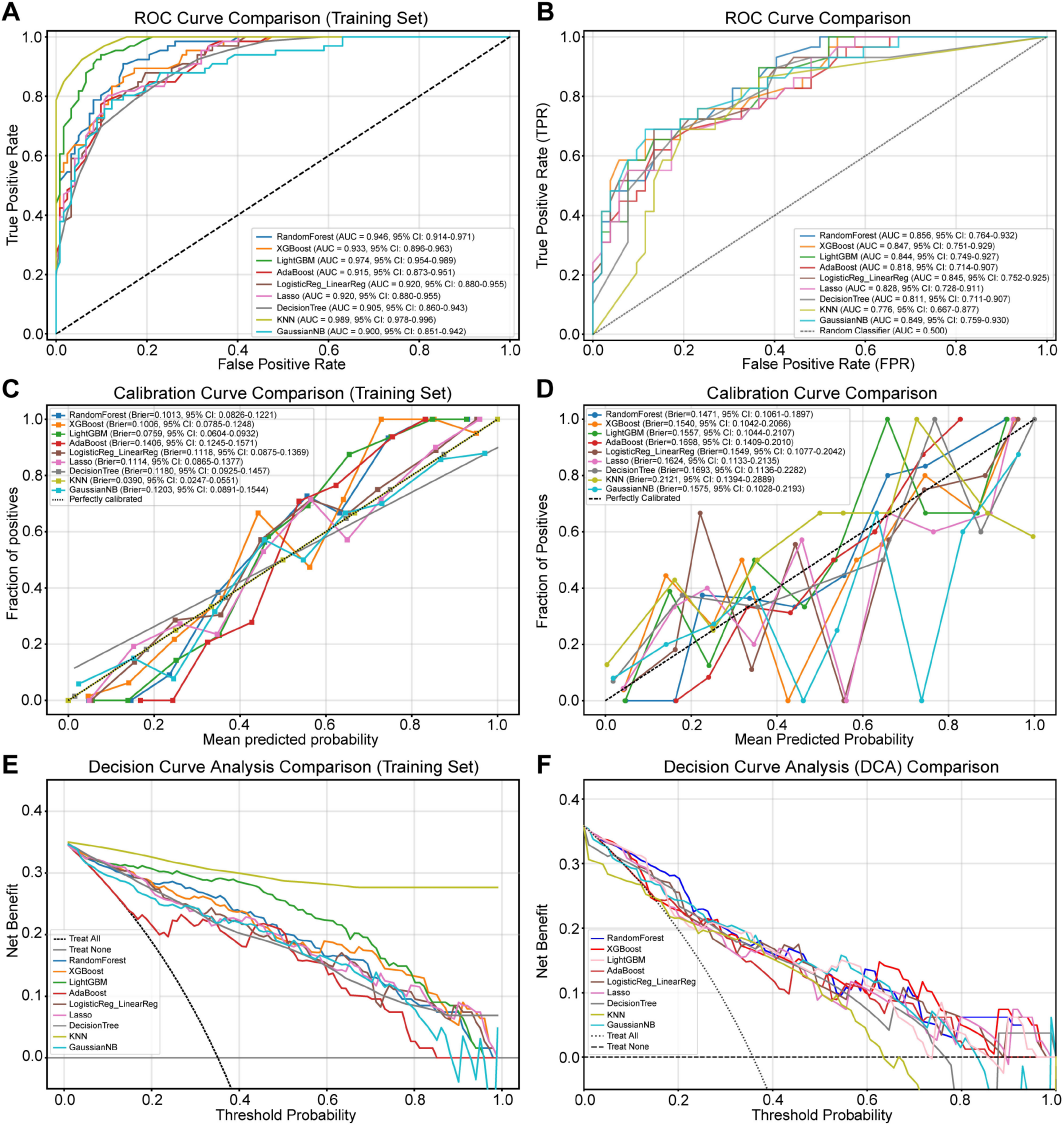
Performance of nine ML models for predicting WD. **(A)** Training ROC curves (*x*-axis = false positive rate, *y*-axis = true positive rate; dashed line = random classifier); each colored curve = one ML algorithm. **(B)** Validation ROC curves; Random Forest achieved the highest AUC of 0.856 (95% CI: 0.764–0.932). **(C)** Training calibration curves (*x*-axis = mean predicted probability, *y*-axis = fraction of positives; dashed line = perfect calibration; Brier score in legend, lower = better). **(D)** Validation calibration curves; Random Forest demonstrated closest agreement with perfect calibration (slope = 1.007, intercept = - 0.002). **(E)** Training DCA (*y*-axis = net benefit, *x*-axis = threshold probability). The solid black line represents “treat none” (net benefit = 0) and the gray dashed line represents “treat all.” **(F)** Validation DCA. Random Forest provided the greatest net benefit across the clinically meaningful threshold range of 0.10–0.40. AUC, area under the curve; ROC, receiver operating characteristic; DCA, decision curve analysis; RF, Random Forest; LR, Logistic Regression; DT, Decision Tree; KNN, K-Nearest Neighbors; NB, Gaussian Naive Bayes.

Based on eight independent predictive factors, we developed nine ML models (Random Forest, AdaBoost, Logistic Regression, Lasso, Decision Tree, KNN, Gaussian Naive Bayes, XGBoost, and LightGBM) and comprehensively evaluated their performance in terms of discrimination, calibration, and clinical applicability. To improve the model’s performance and generalizability, hyperparameter optimization was performed, with detailed results provided in Supplementary Table 6. In the training cohort, Random Forest demonstrated the highest discrimination ability (AUC = 0.946, 95% CI: 0.914–0.971), while Gaussian Naive Bayes had relatively lower accuracy. In the validation cohort, Random Forest (AUC = 0.856, 95% CI: 0.764–0.932) showed the favorable internal performance, while XGBoost, LightGBM, and AdaBoost performed at an intermediate level (Figures 4a,b, Table 2 and Supplementary Tables 7, 8). In addition to AUC, calibration analysis confirmed good consistency between predicted probabilities and actual observed probabilities (Figures 4C,D), and decision curve analysis indicated that Random Forest consistently provided the greatest net clinical benefit across a wide range of threshold probabilities (Figures 4E,F). Further evaluation metrics, including precision-recall (PR) parameters (Supplementary Tables 9, 10 and Supplementary Figure 5), Brier score (Supplementary Tables 11, 12), and metrics based on the confusion matrix such as sensitivity, specificity, predictive value, accuracy, recall, and F1 score, further validated the superior stability of Random Forest compared to other algorithms. A direct comparison of the AUC distributions across models (Supplementary Table 13 and Supplementary Figure 6) further supported these findings. Specifically, the Random Forest model achieved a calibration slope of 1.007 and intercept of −0.002, indicating good agreement between predicted probabilities and observed event rates (Supplementary Figure 7 and Supplementary Table 14). The Brier score was 0.1463 (95% CI: 0.106–0.190). DCA demonstrated that Random Forest provided the greatest net clinical benefit across the clinically meaningful threshold range of 10–40% (Supplementary Figure 8). At the default threshold of 0.50, sensitivity was 62.1% and specificity 86.5%; a lower threshold of 0.25 yielded sensitivity of 86.2% with specificity of 69.2%, which may be more appropriate for screening purposes (Supplementary Figure 9 and Supplementary Table 15).

**TABLE 2:** Discrimination of nine ML models in the training and validation cohorts.

Model	Cohort	Sensitivity	Specificity	Pos Pred value	Neg Pred value	Precision	Recall	F1
Random forest	Training	68.2%	94.3%	86.5%	84.6%	86.5%	68.2%	76.3%
XGBoost	Training	74.2%	89.3%	79.0%	86.5%	79.0%	74.2%	76.6%
LightGBM	Training	81.8%	95.0%	90.0%	90.6%	90.0%	81.8%	85.7%
AdaBoost	Training	75.8%	90.2%	80.6%	87.3%	80.6%	75.8%	78.1%
Logistic regression	Training	66.7%	92.6%	83.0%	83.7%	83.0%	66.7%	73.9%
Lasso	Training	68.2%	91.8%	81.8%	80.6%	81.8%	68.2%	74.4%
Decision tree	Training	69.7%	90.2%	79.3%	84.6%	79.3%	69.7%	74.2%
KNN	Training	84.8%	98.4%	96.6%	92.3%	96.6%	84.8%	90.3%
GaussianNB	Training	72.7%	89.3%	78.7%	85.8%	78.7%	72.7%	75.6%
Random forest	Validation	62.1%	86.5%	72.0%	80.4%	72.0%	62.1%	66.7%
XGBoost	Validation	65.5%	84.6%	70.4%	81.5%	70.4%	65.5%	67.9%
LightGBM	Validation	65.5%	84.6%	70.4%	81.5%	70.4%	65.5%	67.9%
AdaBoost	Validation	62.1%	82.7%	66.7%	79.6%	66.7%	62.1%	64.3%
Logistic regression	Validation	55.2%	86.5%	69.6%	77.6%	69.6%	55.2%	61.5%
Lasso	Validation	55.2%	84.6%	66.7%	77.2%	66.7%	55.2%	60.4%
Decision tree	Validation	62.1%	84.6%	69.2%	80.0%	69.2%	62.1%	65.5%
KNN	Validation	58.6%	82.7%	65.4%	78.2%	65.4%	58.6%	61.8%
GaussianNB	Validation	69.0%	82.7%	69.0%	82.7%	69.0%	69.0%	69.0%

Cohort, dataset partition (Training = model development set, Validation = internal validation set); All metrics (Sensitivity, Specificity, PPV, NPV, Precision, Recall, F1) are in percentage (%) format. Logistic Regression refers to L2-regularized logistic regression with C = 1, and linear regression models were not used for the binary classification task. KNN, K-nearest neighbors; NB, Naïve Bayes.

To further assess model stability across different data-splitting strategies, we performed additional evaluation experiments including five-fold stratified cross-validation, 10-fold stratified cross-validation, 5 × 5 repeated stratified cross-validation, and averaged results from 30 random 70:30 splits. As shown in Supplementary Table 16 and Supplementary Figure 10, Random Forest achieved a mean AUC of 0.885 ± 0.029 (five-fold CV) and 0.877 ± 0.029 (10-fold CV), consistently demonstrating competitive performance across all strategies. The multi-strategy comparison heatmap (Supplementary Figure 11) confirms that Random Forest maintained stable AUC values (range: 0.845–0.885) regardless of the data-splitting approach. Learning curve analysis (Supplementary Figure 12) further demonstrated convergence of training and validation performance with increasing sample size. Extended performance metrics including balanced accuracy, Matthews correlation coefficient (MCC), and Cohen’s Kappa are provided in Supplementary Table 17, with Random Forest achieving an MCC of 0.504 and Kappa of 0.501 in the validation cohort, confirming moderate-to-good agreement beyond chance.

### Random Forest as the best-performing predictive model and SHAP-based interpretability analysis

**FIGURE 5:**
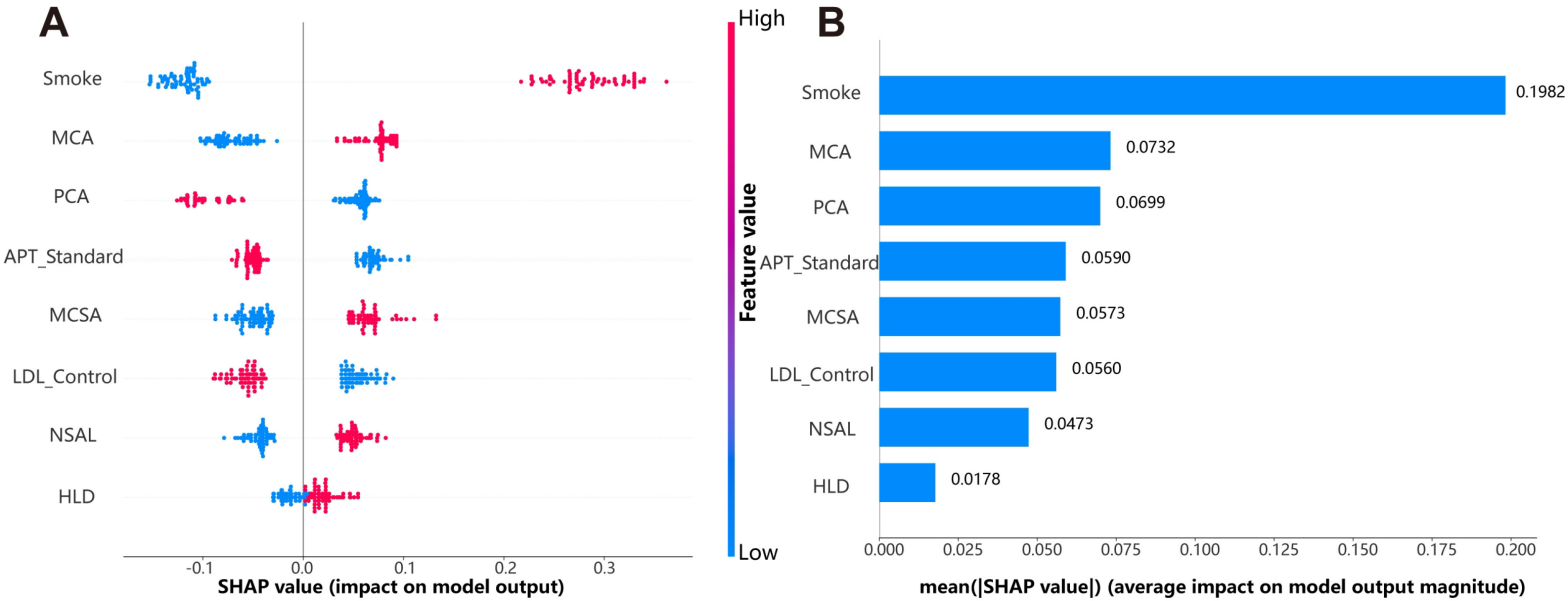
SHAP (Shapley Additive Explanations)-based feature importance analysis for the best-performing random forest model. **(A)** Beeswarm summary plot displaying the SHAP values (*x*-axis, impact on model output) for each of the eight predictive features (*y*-axis, ordered by overall importance from top to bottom). Each dot represents one patient in the training cohort (*n* = 188). The color gradient indicates the original feature value: red = high (feature present/value = 1), blue = low (feature absent/value = 0). Points to the right of zero indicate positive contributions toward WD prediction; points to the left indicate negative contributions. Smoking history exhibited the strongest positive impact, with high feature values (smoker) consistently pushing predictions toward WD. **(B)** Bar chart of mean absolute SHAP values, representing the average magnitude of each feature’s contribution to the model output across all training samples. SHAP, Shapley Additive Explanations; MCA, middle cerebral artery; PCA, posterior cerebral artery; APT, antiplatelet therapy; LDL-C, LDL cholesterol control.

Given that Random Forest demonstrated consistent advantages across multiple evaluation metrics, we selected Random Forest as the best-performing model for predicting WD. The Random Forest classifier exhibited encouraging performance, with a balanced accuracy of 85.1% in the training cohort and 77.8% in the validation cohort. In the training set, the model’s overall accuracy was 85.1%, with a sensitivity of 68.2%, specificity of 94.3%, precision of 86.5%, recall of 68.2%, and an F1 score of 76.3%. Similar results were obtained in the validation set, with an accuracy of 77.8%, sensitivity of 62.1%, specificity of 86.5%, precision of 72%, recall of 62.1%, and an F1 score of 66.7% (Supplementary Figure 13). These results indicate minimal performance fluctuation across resampled datasets, supporting the stability of the Random Forest model in potential clinical applications. Overall, the Random Forest model demonstrated stable discriminative ability, maintaining high sensitivity and specificity in both cohorts, with good consistency between predicted outcomes and actual results. To enhance the interpretability of the Random Forest model, we employed the Shapley Additive Explanations (SHAP) method to quantify the contribution of each predictive variable to the model’s output. As shown in Figure 5, smoking history, MCA and PCA were the most significant contributors, followed by standardized antiplatelet therapy, maximum cross-sectional area of the stroke, achieving LDL target with oral statins, the number of stroke-affected layers, and hyperlipidemia. It should be noted that SHAP values quantify each feature’s contribution to the model’s prediction output and should be interpreted as measures of predictive importance, not biological causality. SHAP interaction values represent model-level associations rather than confirmed biological interaction mechanisms. Subgroup analysis confirmed consistent model performance across age and sex subgroups (Supplementary Figure 14). Predictor correlation analysis confirmed absence of severe multicollinearity among the eight selected features (|r| < 0.7 for all pairs; Supplementary Figure 15).

**FIGURE 6:**
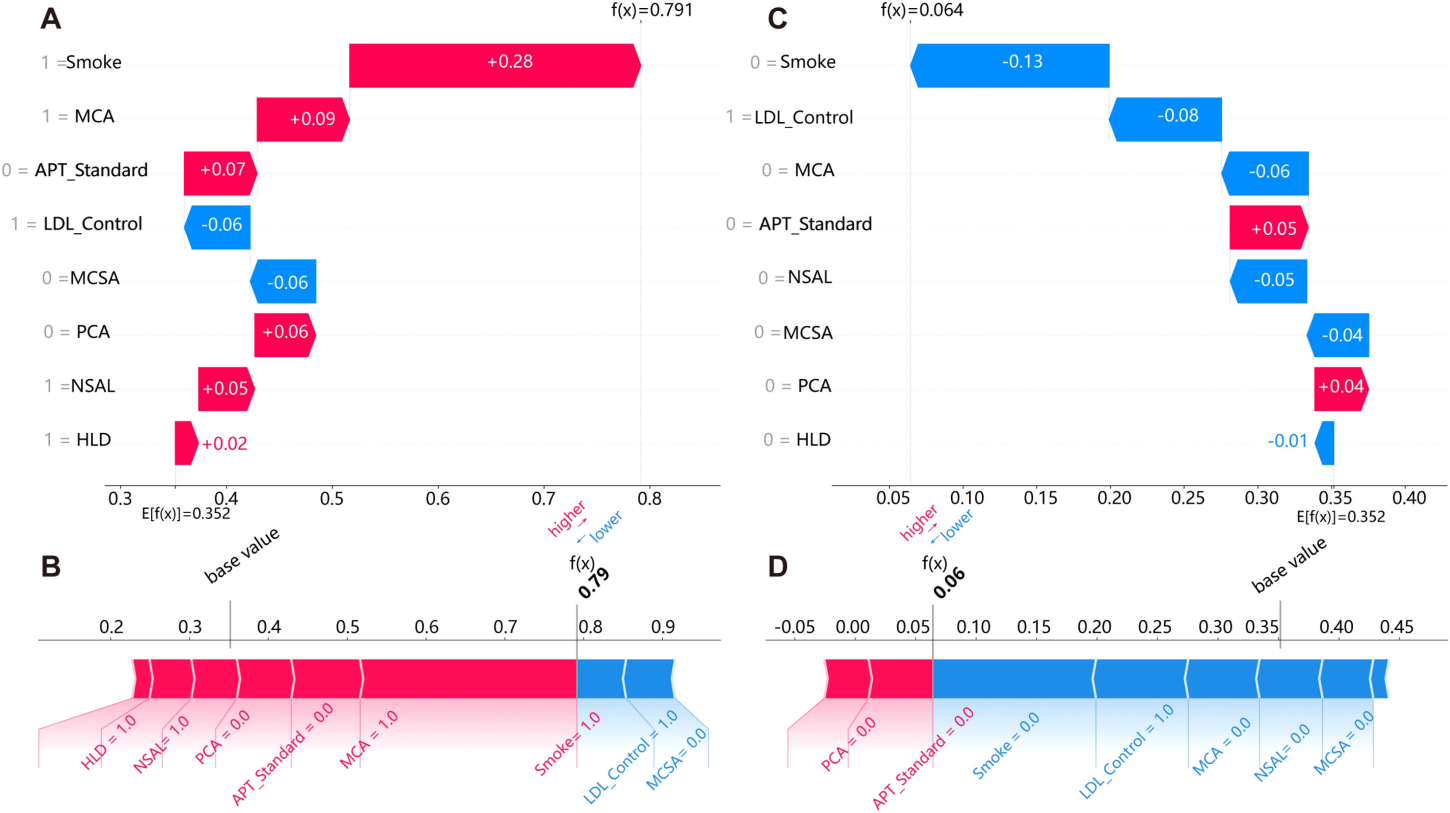
Representative individual-level SHAP analysis plot. **(A)** Waterfall plot for a representative WD-positive case [predicted probability *f*(x) = 0.791]. Each horizontal bar represents the SHAP contribution of one feature, with red bars indicating positive contributions (pushing toward WD prediction) and blue bars indicating negative contributions (pushing away from WD prediction). The numerical label on each bar shows the signed SHAP value ( ± ). Feature values are shown on the left (e.g., “1 = smoke” means the patient is a smoker). The base value E[*f*(X)] = 0.352 represents the average prediction across all training samples. Smoking history (+0.28) was the dominant positive contributor. **(B)** Corresponding force plot for the same WD-positive case, showing the cumulative effects of all features in a horizontal format. Red segments push the prediction rightward (toward WD); blue segments push leftward (away from WD). **(C)** Waterfall plot for a representative non-WD case [*f*(x) = 0.064]. Non-smoking (- 0.13), non-MCA involvement (- 0.06), and LDL-C control (- 0.08) were the dominant protective contributors. **(D)** Force plot for the same non-WD case. SHAP, Shapley Additive Explanations; WD, Wallerian degeneration; *f*(x), predicted probability; E[*f*(X)], expected prediction. Feature abbreviations as defined in Figure 5.

To provide more intuitive clinical interpretation beyond global feature importance, we further presented outcome-stratified SHAP visualizations in Figure 6, including representative force and waterfall plots for the WD group (positive samples) and the non-WD group (negative samples). These plots clearly illustrate the direction and magnitude of each feature’s contribution, showing how key predictive factors collectively drive individual predictions toward higher or lower risk of WD. To further enhance clinical interpretability, we also provide SHAP dependence plots for key predictive variables (Supplementary Figure 16). By analyzing the SHAP values of each feature, we can identify the most important features for the model’s predictions, such as smoking history, MCA, and PCA, which play a key role in predicting the progression of WD. In addition, we plotted a series of SHAP-based visual analysis plots. The SHAP summary plot (Supplementary Figure 17A) shows the feature importance, highlighting the most influential variables, with smoking having the highest contribution. The mean SHAP value plot (Supplementary Figure 17B) shows that features such as smoking and MCA have the highest average SHAP values. The SHAP heatmap (Supplementary Figure 17C) visualizes the individual-level contribution patterns of the final selected predictive factors in the best-performing Random Forest model. The color intensity indicates the impact of each feature on the model output, with smoking and MCA showing the most consistent influence across different instances. The SHAP effects and interaction network graph (Supplementary Figure 17D) illustrates the interactions between features and their collective impact on the model output, with features like smoking and MCA showing strong interactions.

The feature importance ranking further emphasizes the multifactorial and interdependent nature of diagnosing WD, integrating demographic factors, coagulation indicators, and imaging features to build a coherent risk assessment framework. After considering their individual impact on the model’s output, we also examined how they interact to collectively influence the predicted outcome (Supplementary Figure 18 and Supplementary Table 18). From the table, we can see that the top five strongest feature interactions were the interaction between maximum cross-sectional area of the stroke and MCA (strength: 0.0091), the interaction between smoking history and MCA (strength: 0.0090), the interaction between smoking history and hyperlipidemia (strength: 0.0089), the interaction between smoking history and PCA (strength: 0.0085), and the interaction between smoking history and the number of stroke-affected layers (strength: 0.0085). These interaction strengths indicate that the combined effects of the maximum cross-sectional area of the stroke, smoking history, and these features have a significant impact on the model’s predictions, especially regarding the correlation between vascular health and stroke severity. The Random Forest model not only demonstrates internally favorable predictive performance with reasonable internal consistency within the study population, and maintains enhanced interpretability through SHAP analysis, providing a preliminary framework for early risk assessment of WD that requires external validation before clinical deployment.

## Discussion

So far, ML models for predicting WD after IS remain relatively limited. This study proposes and validates an interpretable ML prediction model to address this significant clinical challenge. We constructed multiple ML models based on eight key variables selected through LASSO regression from a wide range of clinical features. Among the tested algorithms, the Random Forest model demonstrated internally favorable predictive performance with reasonable internal consistency within the study population. The main findings of our study can be summarized as follows: (1) Random Forest demonstrated superior predictive performance, achieving an AUC of 0.856 in the validation cohort; (2) SHAP analysis identified smoking history, hyperlipidemia, standardized antiplatelet therapy, achieving LDL target with oral statins, maximum cross-sectional area of the stroke, MCA, PCA, and the number of stroke-affected layers as the most influential predictive factors. Compared to previous representative ML studies in IS, which mainly focused on broader endpoint events (such as mortality or long-term functional outcomes), this study uses strict WD occurrence as an endpoint and integrates multimodal predictive indicators, which may partly explain the differences in performance metrics and the selection of the best-performing algorithm across studies (32). Its kernel-based framework has a unique advantage in capturing nonlinear interactions between risk factors, thereby overcoming the limitations of traditional regression models.

Our model identified smoking history as one of the strongest predictors of WD occurrence. Harmful substances in cigarettes (such as nicotine, carbon monoxide, free radicals, etc.) have certain toxic effects on the nervous system. Through mechanisms such as oxidative stress and free radical damage, vasoconstriction and reduced blood flow, neuroinflammatory responses, and blood-brain barrier damage, smoking further exacerbates ischemic damage, leading to neuronal destruction, myelin injury, and triggering WD. Experimental studies have shown that smoking enhances PLT aggregation and leads to arterial vasoconstriction by increasing sympathetic nervous activity, thereby increasing the risk of stroke (33). At the same time, nicotine promotes thrombin generation while weakening fibrinolysis, leading to the formation of unstable thrombi (34, 35). Recent animal experimental evidence indicates that long-term tobacco exposure triggers a cascade of damage initiated by cerebrovascular dysfunction, manifested as sustained reduction in cerebral blood flow, disruption of the blood-brain barrier, and oxidative neuronal injury, thereby leading to the onset and progression of ischemic stroke (36). Regarding variable measurement timing, the intended prediction scenario is risk assessment during follow-up in patients with prior IS, not acute admission prediction. This model is designed exclusively for follow-up monitoring and should not be applied at the point of acute admission. Demographic factors were pre-existing, imaging features were measured during the acute phase, and treatment adequacy reflects ongoing management (Supplementary Figure 19). A sensitivity analysis excluding the two treatment-related variables (APT Standard and LDL-C Control) yielded a validation AUC of 0.821, confirming that demographic and imaging features alone provide meaningful prediction (Supplementary Table 19).

Imaging characteristics, such as the responsible blood vessels, size, and layers of the stroke, are also key predictive factors. Among the different responsible blood vessels for the stroke, MCA and PCA have the highest risk for WD, which may be related to corticospinal tract (CST) axonal damage. The MCA supplies blood to the frontal lobe, parietal lobe, lateral temporal lobe, insula, and basal ganglia (via the lenticulostriate arteries), especially including the posterior limb of the internal capsule, a key pathway of the CST. When an MCA infarction involves the precentral gyrus, corona radiata, posterior limb of the internal capsule, or deep structures of the basal ganglia, it can directly damage CST axons, leading to anterograde degeneration of distal axons, which then results in secondary atrophy in the brainstem (midbrain, pons, medulla), i.e., typical WD. Previous studies have shown that the MCA is the most common site of stenosis (37), followed by the PCA. In our model, We note that MCA and PCA, while retained as predictors by the LASSO algorithm and contributing meaningfully to the Random Forest model’s predictions (as demonstrated by SHAP analysis), did not achieve statistical significance in the multivariate logistic regression. This discrepancy is not uncommon and reflects the fundamental difference between parametric regression models (which test individual coefficient significance under linearity assumptions) and ensemble ML algorithms (which can leverage subtle, nonlinear, and interactive contributions of features). The Random Forest model captures these complex contributions through recursive partitioning and aggregation, which is not reflected in individual regression *p*-values. Therefore, while MCA and PCA should not be described as statistically significant independent predictors in the regression framework, their inclusion in the ML model is justified by their LASSO selection, SHAP importance, and established biological relevance. The MCA as the responsible blood vessel for WD showed particularly significant risk, consistent with the findings of Gupta et al. This study, through longitudinal DTI research, found that large infarctions in the MCA region could secondarily cause WD in the corpus callosum (38). Additionally, the larger the infarction area, the higher the probability of WD. This may be due to the larger lesion area involving more neural conduction pathways, making it easier for demyelination and WD to occur after injury. A retrospective analysis of 1,809 cranial CT scans found that large hemispheric infarctions involving most of the motor cortex often showed clear WD in the ipsilateral brainstem, while small infarctions ( ≤ 2.5 cm) or lacunar infarctions usually did not lead to obvious secondary degeneration (39). Furthermore, with the advancement of imaging, the descending pyramidal tract (DPT) damage can be visualized through FLAIR and DWI sequences, and the directionality and integrity of white matter fibers can be tracked using DTI to quantitatively monitor changes in nerve fiber bundles (40). When only 1–2 layers are affected, it usually does not cause atrophy or mild atrophy of the midbrain cerebral peduncles.

Laboratory biomarkers further provide mechanistic explanations. Whether PLT and LDL levels are within the target after standardized treatment, along with hyperlipidemia, is significantly related to the occurrence of WD. Standardized antiplatelet therapy can effectively control stroke risk factors and prevent further brain tissue damage (41). High PLT activity may exacerbate vascular occlusion, leading to cerebral infarction, which may then promote WD in the infarcted area. Hyperlipidemia refers to abnormal elevation of lipids (especially total cholesterol, triglycerides, and LDL) in the blood, with low-density lipoprotein being a key factor in the development of atherosclerosis. High levels of both LDL and HDL are associated with large artery atherosclerotic stroke (42). Prolonged high LDL can promote endothelial damage, plaque formation, and vascular blockage, increasing the risk of cerebral infarction, leading to more severe CST damage and WD. Previous studies have also reported higher recurrence rates of IS caused by atherosclerosis (43). A single-center randomized controlled trial including 120 patients with 50–99% symptomatic intracranial artery atherosclerotic stenosis aimed to compare the effects of high-intensity and low-intensity atorvastatin treatment. After 52 weeks of follow-up, the results showed that the high-intensity atorvastatin group had a significantly lower cumulative probability of recurrent cerebrovascular events compared to the low-intensity group (44). These findings are consistent with those of Yu et al. (45). In our cohort, achieving LDL-C control targets was inversely associated with WD (OR = 0.212, a protective effect), while higher PLT levels reflected prothrombotic risk, consistent with their established roles in the atherosclerotic process. The inclusion of treatment adequacy variables (APT Standard and LDL-C Control) is appropriate within the follow-up monitoring framework, as these represent modifiable risk factors reflecting the quality of secondary prevention (Supplementary Table 23 and Supplementary Figure 26). Therefore, we are more inclined to interpret these as biomarkers that predispose to WD and can be used for early risk assessment, rather than a new mechanistic discovery. High PLT activity and high LDL levels increase coagulation, thereby increasing the risk of IS recurrence and WD occurrence.

We agree that core ML components, such as model types, internal validation strategies, and performance evaluation metrics, have been widely adopted in the relevant literature. Additionally, interpretability tools like SHAP and nomogram-style clinical translation methods have also been reported. For example, Zhang et al. constructed a machine learning framework combining LASSO and Boruta dual feature selection strategies with SHAP interpretability analysis in their study on predicting the risk of swallowing disorders after acute ischemic stroke in the elderly, reflecting the trend of clinical prediction modeling evolving from single algorithms to integrated explanatory methods (46). It is worth noting that while Random Forest was identified as the best-performing model in our study, its performance was similar to that of other algorithms (such as XGBoost and neural networks). This phenomenon does not indicate a fundamental difference between algorithms but rather reflects the significant impact of data distribution, feature composition, and population heterogeneity on model performance in stroke-related complication prediction tasks (47). Differences between studies typically arise from multiple factors (32), including the definition of endpoint events and time windows, the severity and composition of the study population, the availability of feature variables (especially imaging variables), sample size and class imbalance, data preprocessing and missing value imputation strategies, as well as hyperparameter tuning and validation processes. In this context, our research approach was to compare multiple mature algorithms under a unified feature set and evaluation framework and select the best-performing model based on the clinically actionable endpoint—the occurrence of WD. It is important to note that SHAP values quantify feature contributions to the model’s output and reflect predictive importance, not causal or pathophysiological significance. While we discuss biological plausibility of the identified risk factors in the context of existing literature, the cross-sectional observational design of our study precludes causal inference. The SHAP-based insights should be viewed as hypothesis-generating and as evidence that the model’s predictions align with clinically and biologically plausible patterns, rather than as establishing new mechanistic pathways.

This study’s added value lies not in discovering entirely new risk factors but in quantifying and integrating multifactorial risks for a clinically actionable endpoint—the occurrence of WD, which remains an under-studied clinical complication with significant prognostic implications. ML allows for the integration of heterogeneous data from different fields, including imaging, coagulation, and metabolic-related indicators, while capturing nonlinear contributions that are challenging to represent through linear models. (1) We employed a rigorous feature selection strategy and compared nine ML algorithms. Within the scope of our internal data, Random Forest emerged as the best-performing model, though its clinical applicability would require prospective external validation in independent cohorts. (2) To enhance interpretability and clinical relevance, we focused not only on the model’s discriminative ability but also on calibration and decision analysis net benefit, linking predictions to biologically plausible patterns using SHAP analysis. This approach provides a transparent framework for early, risk-stratified monitoring and intervention strategies. While experienced clinicians can identify these risk factors, integrating them into a consistent, patient-specific risk estimate can involve subjectivity and vary between practitioners. Therefore, our model aims to assist, not replace, clinical judgment by providing objective, reproducible risk assessments, demonstrating its potential for clinical applicability across different decision thresholds. Recent work by Yang et al. (48) demonstrated that deep learning models integrating DWI-MRI and clinical features achieved superior prognostic prediction for acute ischemic stroke, with the clinical-deep learning fusion model reaching an AUC of 0.925 in internal validation and 0.937 in external validation (48). Our study extends this paradigm by targeting WD as a more specific post-stroke complication, using readily available clinical variables that may enhance practical applicability in routine settings.

To contextualize our findings, we compared our model’s performance with prior ML-based prediction studies targeting stroke-related endpoints (Supplementary Table 20 and Supplementary Figure 20). Our Random Forest model achieved a validation AUC of 0.856, which is comparable to or exceeds the performance of previous studies: Fast et al. (AUC = 0.820 for IS outcome prediction), Tanioka et al. (AUC = 0.810 for hematoma expansion), Zhang and Zhao (AUC = 0.847 for dysphagia prediction), and Abujaber et al. (AUC = 0.830 for 90-day prognosis). Notably, our study is the first to specifically target WD as a clinical endpoint using an interpretable ML framework, and demonstrates favorable performance despite using only eight predictive features, suggesting that a parsimonious feature set can achieve clinically meaningful prediction accuracy when guided by domain knowledge and rigorous feature selection.

However, there are still several limitations to consider. (1) despite statistical adjustments, residual confounding factors are inevitably present in observational studies. (2) this study is based on a single-center population in China, and its external applicability needs to be validated in different racial and healthcare settings. (3) while the model performed well in internal validation, it still needs to be directly compared with clinician assessments before clinical implementation and validated through large-scale, multi-center prospective studies. Additionally, the modest sample size (*n* = 269, 95 events) may limit the precision of performance estimates. Although the model demonstrated stable performance across multiple internal validation strategies (holdout, 5–fold CV, 10-fold CV, 5 × 5 repeated CV, and 30-random splits), these remain forms of internal validation and do not establish external generalizability. The model should be regarded as a preliminary prediction tool requiring multicenter external validation, prospective testing, and comparison with clinician judgment before clinical use.

Future research should further strengthen the clinical translational value of the model. Although SHAP provides sample-level explanations, this study mainly focused on model development and retrospective validation. In subsequent studies, we plan to incorporate individual-level longitudinal trajectory data (e.g., imaging and laboratory indicators changing over time) to explore how patients’ dynamic status affects the risk of WD occurrence. Additionally, we plan to conduct prospective, multi-center validation and implementation studies to evaluate the practical impact of model-assisted decision-making in real clinical settings. If the model is externally and prospectively validated, it could serve as a risk calculation tool during follow-up visits (e.g., integrated with electronic medical record systems) to support early risk assessment and guide individualized decisions, such as determining the timing of repeat MRIs and monitoring intensity, particularly prioritizing early imaging exams and closer monitoring for high-risk patients.

## Conclusion

In this study, we developed and validated a promising, internally validated ML model—Random Forest—to predict the occurrence of WD after IS. The model showed favorable internal discrimination and calibration, and with the integration of SHAP analysis, it demonstrated promising clinical interpretability. By incorporating key demographic, imaging, and laboratory features, the model provides a preliminary internally validated framework that warrants external and prospective validation before clinical implementation. Its translational potential lies in optimizing personalized management strategies and guiding timely interventions, thereby improving the prognosis of this patient population.

## Data Availability

The raw data supporting the conclusions of this article will be made available by the authors, without undue reservation.
