# Supplementary material for: Establishment of a machine learning prediction model for Wallerian degeneration after ischemic stroke
Source: Front Med (Lausanne). 2026 Jun 30;13:1841202. doi: 10.3389/fmed.2026.1841202 (PMC13365044; doi:10.3389/fmed.2026.1841202)
Supplement: Supplementary file 1 [file Supplementary_file_1.DOCX]

**Figure S1 Participant screening, enrollment, and cohort allocation flow diagram.**

Flow diagram illustrating the participant selection process in tabular/visual format. A total of 308 IS patient records were screened, of which 39 were excluded: 10 for newly diagnosed IS, 15 for WD caused by non-ischemic stroke, and 14 for incomplete mRS/MMSE/MRI assessment. The remaining 269 patients were randomly allocated to the training cohort (n = 188, 70%) and validation cohort (n = 81, 30%) using stratified random sampling. The WD incidence was balanced between cohorts (35.1% vs. 35.8%), confirming effective stratification. Nine ML algorithms were developed and compared, with RandomForest identified as the optimal model (AUC = 0.856, 95% CI: 0.764–0.932).


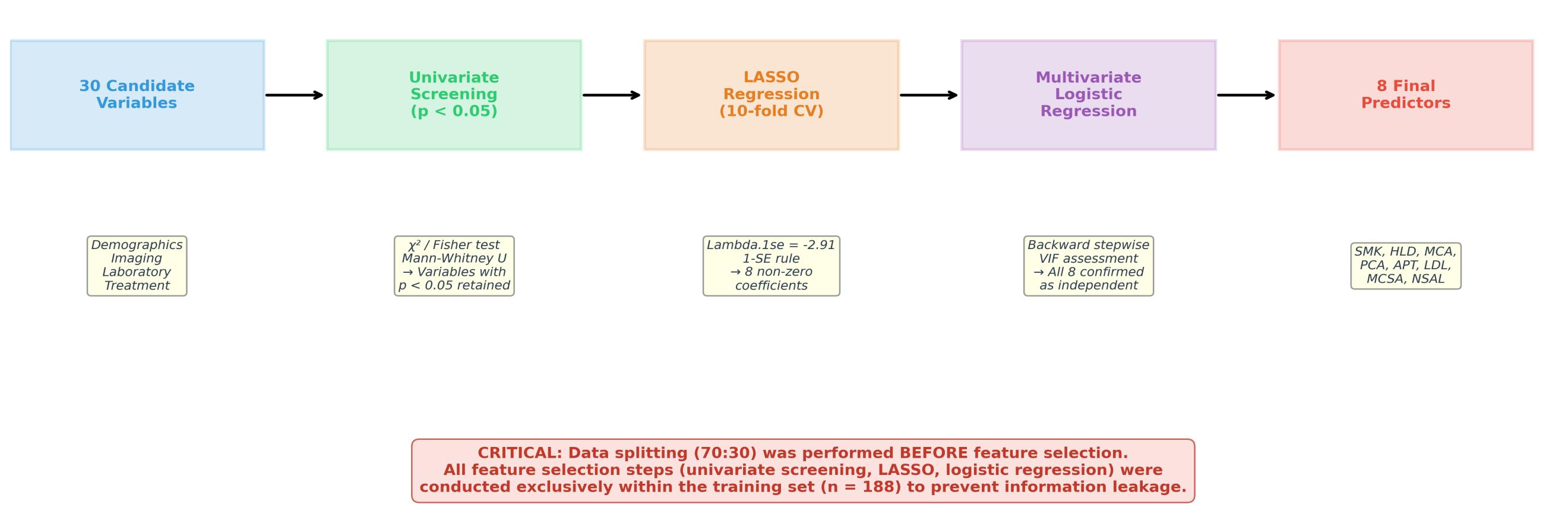


**Figure S2 Feature selection workflow diagram.**

Sequential pipeline from 30 candidate variables through univariate screening (χ²/Fisher, p < 0.05), LASSO logistic regression (10-fold CV, λ.1se = −2.91), and multivariate logistic regression to 8 final predictors. Data splitting (70:30) was performed before all feature selection steps to prevent information leakage.


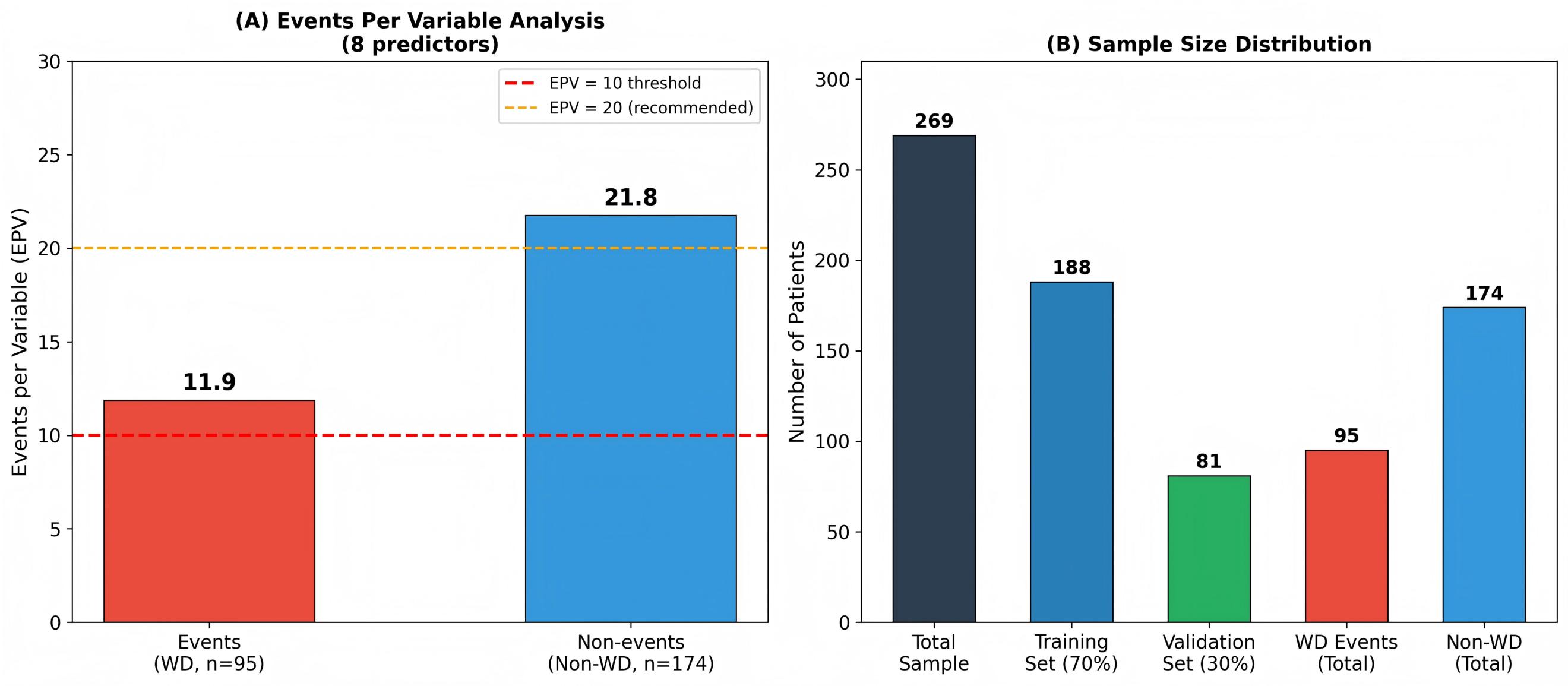


**Figure S3 Events per variable (EPV) analysis and sample size distribution.**

(A) EPV for WD events (n = 95, EPV = 11.9) and non-WD events (n = 174, EPV = 21.8). Red and orange dashed lines indicate EPV thresholds of 10 and 20, respectively. (B) Distribution of sample sizes across total cohort, training set, validation set, and outcome groups.


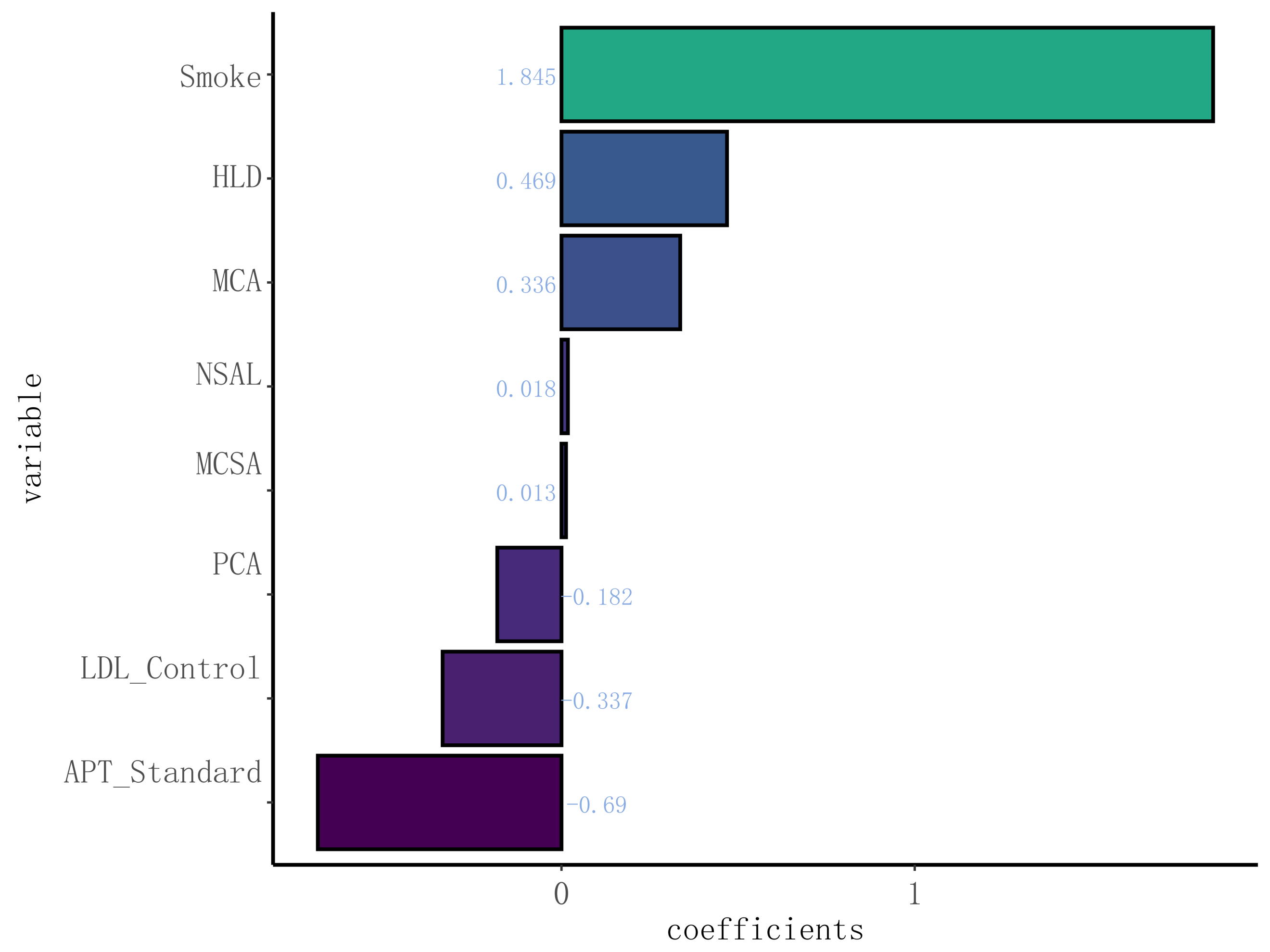


**Figure S4 Bar plot of the LASSO regression coefficients for predictive variables in the ischemic stroke cohort.** The length of each bar represents the magnitude of the corresponding variable's coefficient, and the sign of the coefficient indicates the direction of the relationship between the variable and the outcome (positive or negative).


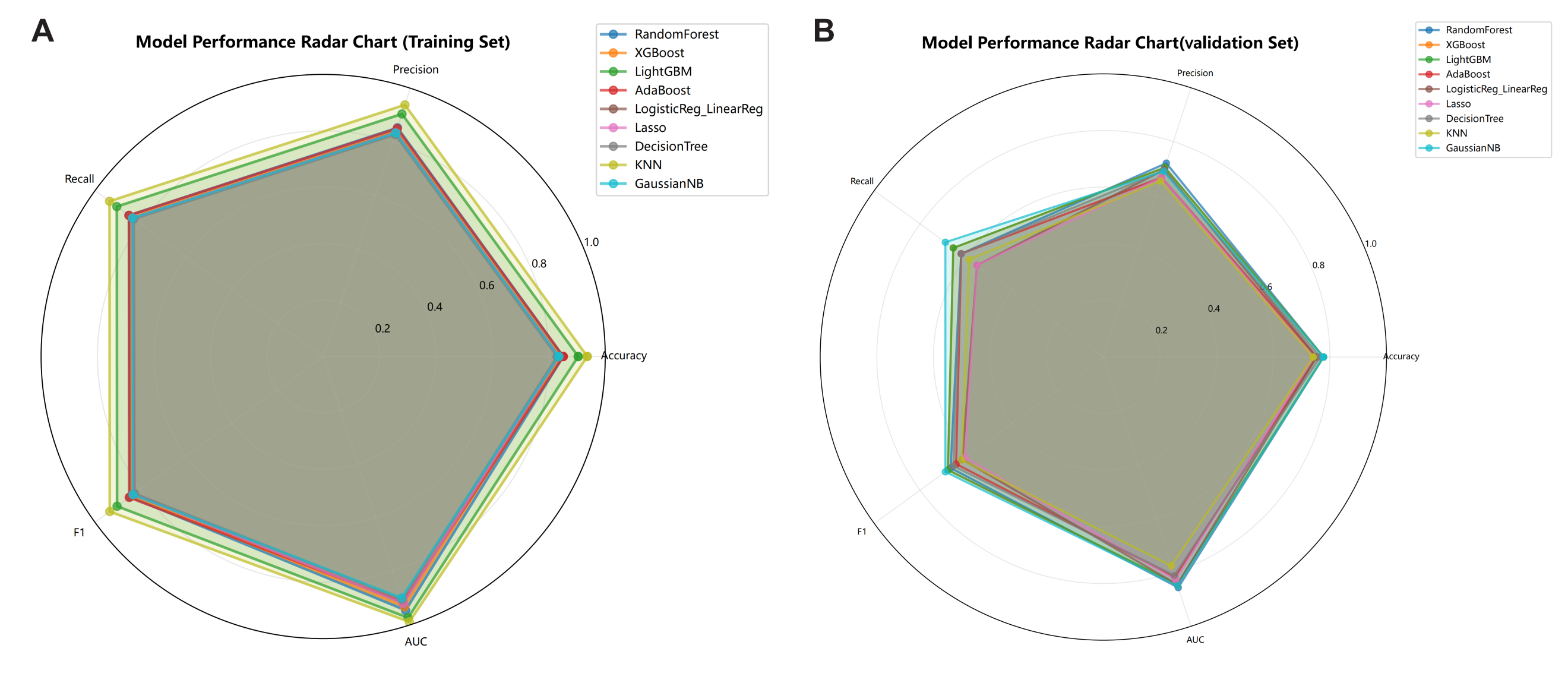


**Figure S5 Radar charts of model performance on the training and validation sets.**

(A) shows model performance on the training set, where RandomForest demonstrates superior performance across multiple evaluation metrics (such as AUC, precision, recall, F1 score, etc.). (B) illustrates model performance on the validation set, where RandomForest maintains strong generalization ability.


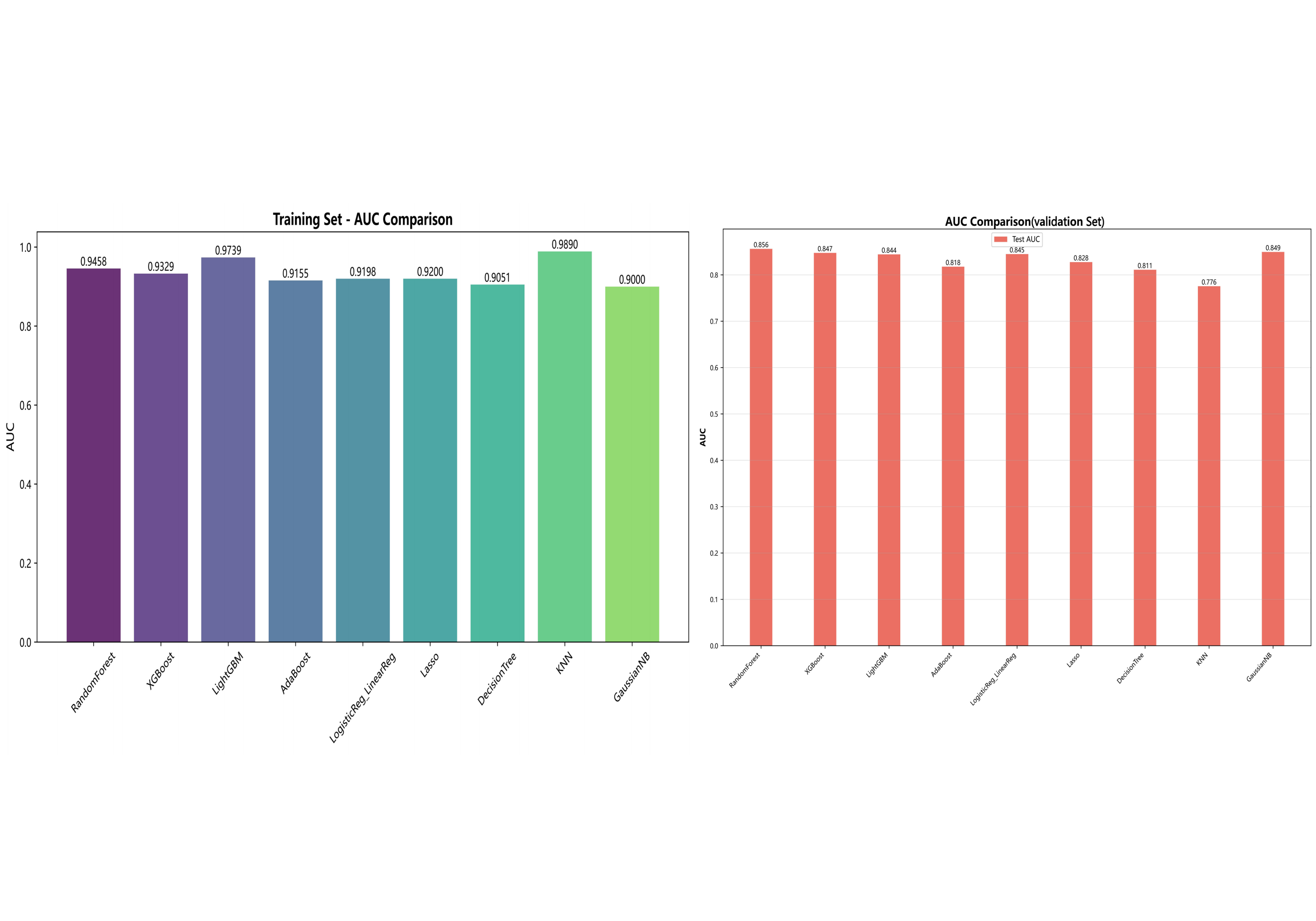


**Figure S6 Comparative AUC distributions of nine ML models.** (A) Bar plots of AUC values in the training cohort. (B) Bar plots of AUC values in the validation cohort.


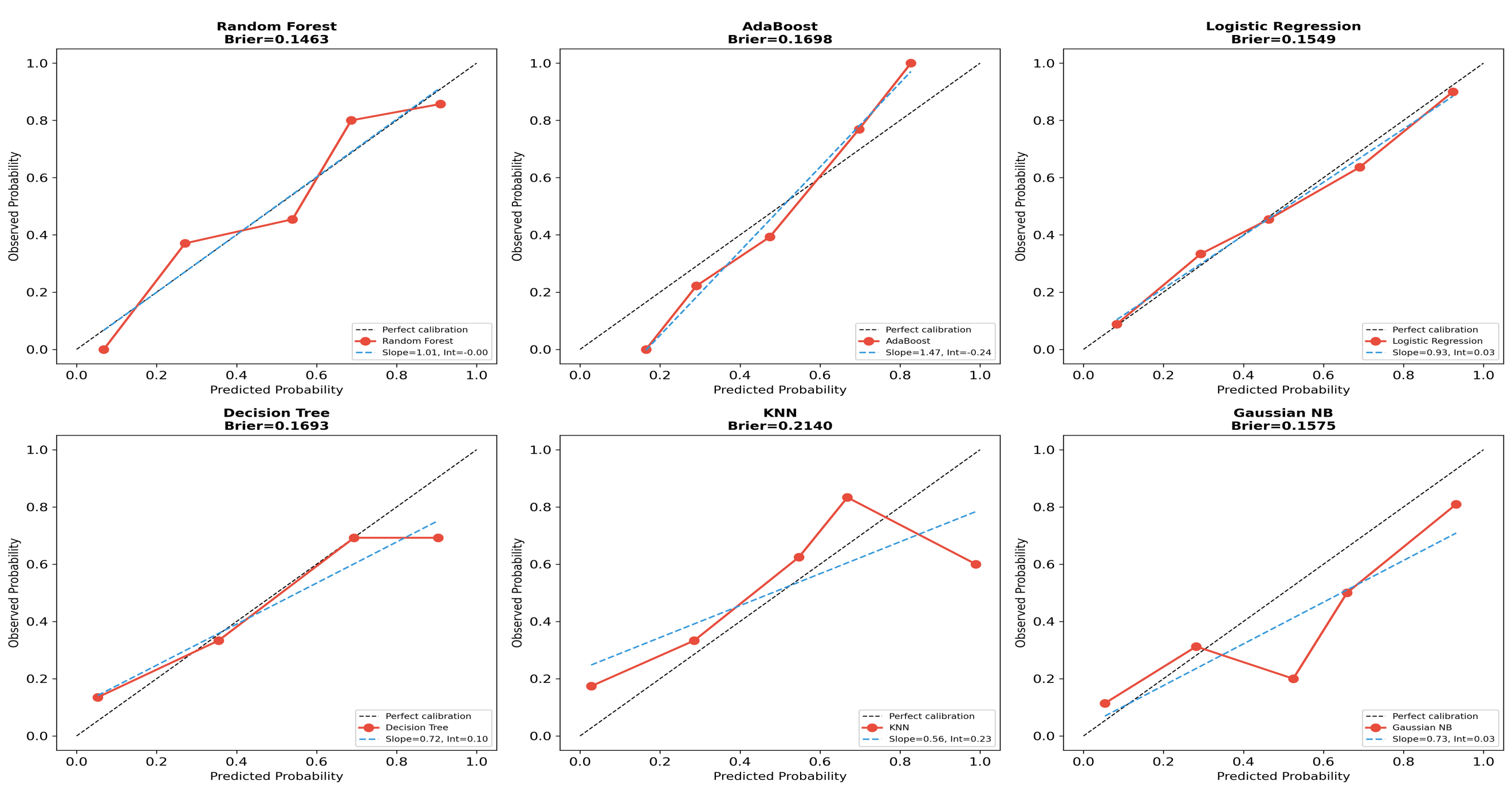


**Figure S7 Calibration analysis with slope, intercept, and Brier scores for six ML models (validation cohort).**

Each panel shows observed versus predicted probability across five calibration bins. Black dashed line = perfect calibration; red circles = observed data; blue dashed line = fitted calibration regression. Random Forest demonstrated the best calibration (slope = 1.007, intercept = −0.002, Brier = 0.1463).


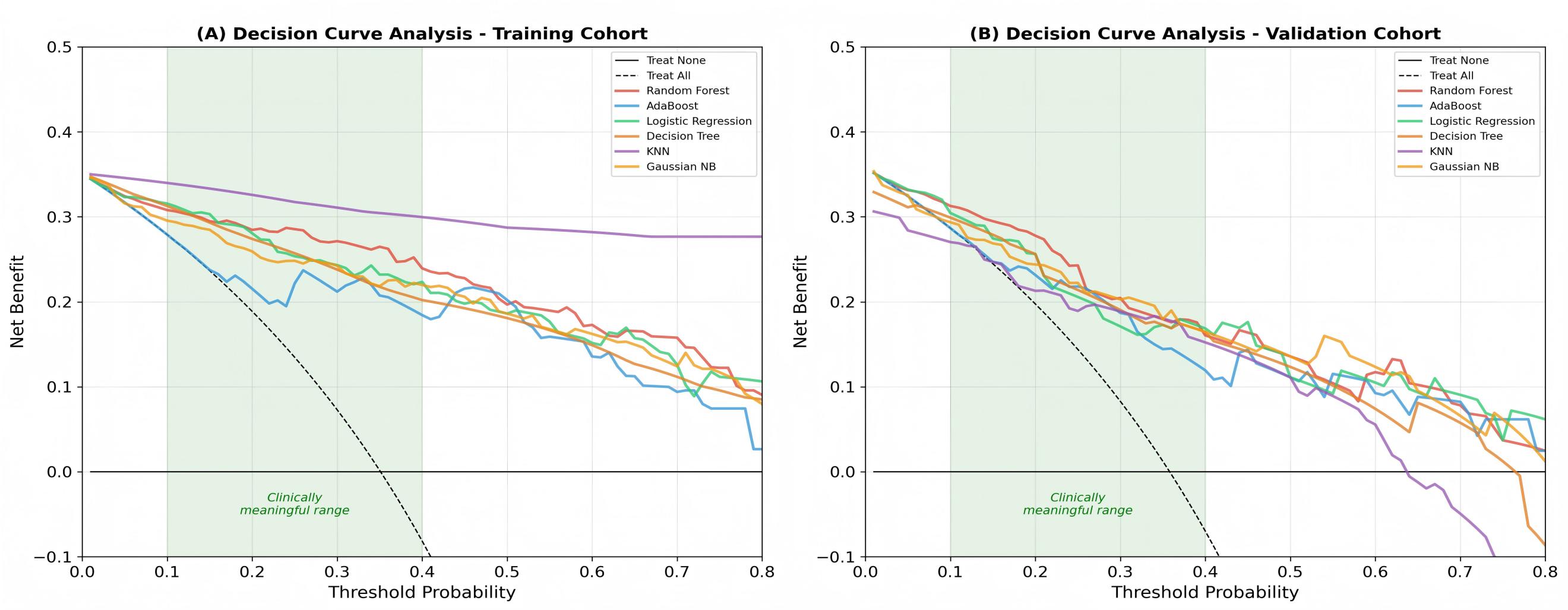


**Figure S8 Decision curve analysis (DCA) with clinically meaningful threshold range.** (A) Training cohort. (B) Validation cohort. Green shaded region (10%–40%) indicates the clinically meaningful threshold range. Random Forest (red) consistently provided the highest net benefit compared to other models and the treat-all/treat-none strategies.


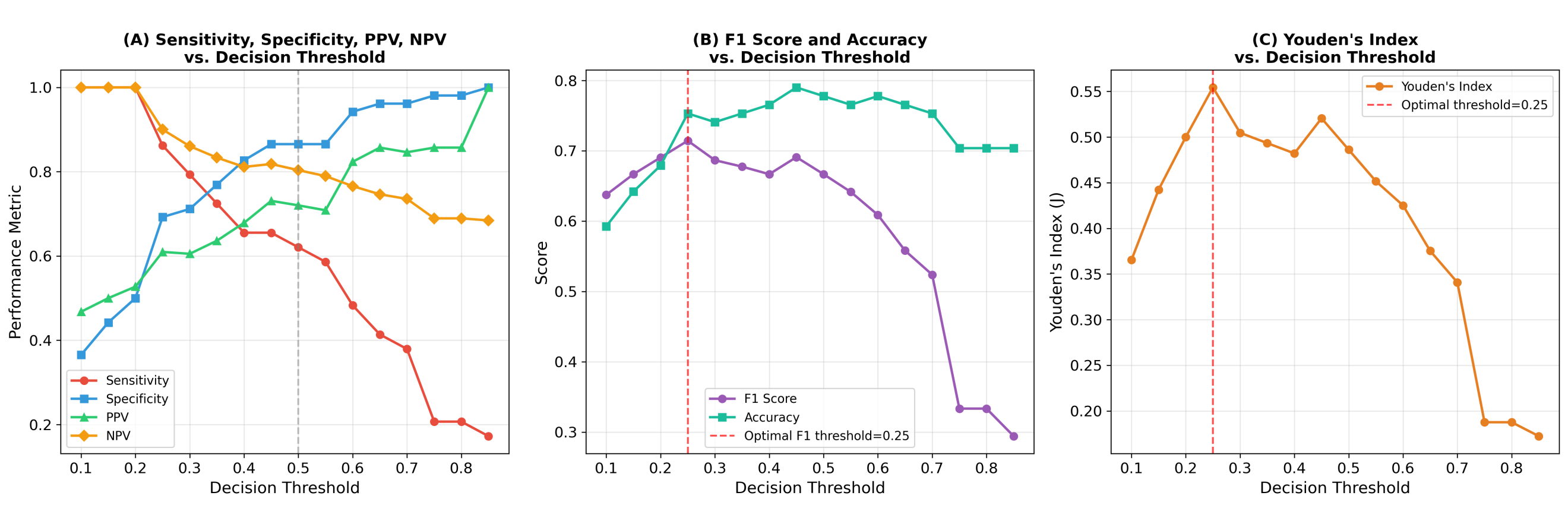


**Figure S9 Threshold sensitivity analysis for the Random Forest model (validation cohort).** (A) Sensitivity (red), specificity (blue), PPV (green), and NPV (orange) across decision thresholds. (B) F1 score and accuracy versus threshold; optimal F1 at threshold = 0.25. (C) Youden’s Index (J = sensitivity + specificity − 1); optimal J = 0.554 at threshold = 0.25.


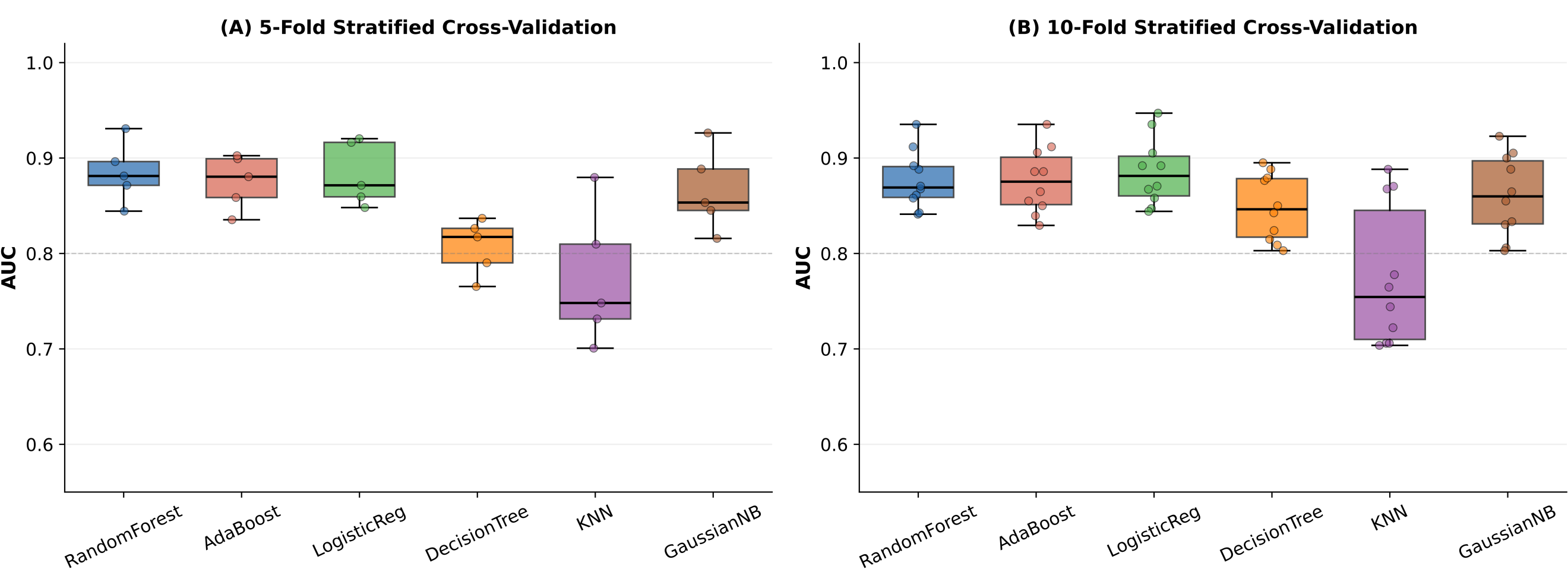


**Figure S10 Cross-validation AUC distributions for six ML models.**

(A) Box plots of AUC values from 5-fold stratified cross-validation. (B) Box plots of AUC values from 10-fold stratified cross-validation. Individual fold-level AUC values are overlaid as scatter points. RandomForest exhibited consistently high AUC values with relatively low variance across both cross-validation strategies (5-fold: AUC = 0.885 ± 0.029; 10-fold: AUC = 0.877 ± 0.029), demonstrating robust generalizability. The dashed horizontal line indicates the AUC = 0.80 threshold commonly considered clinically acceptable for predictive models.


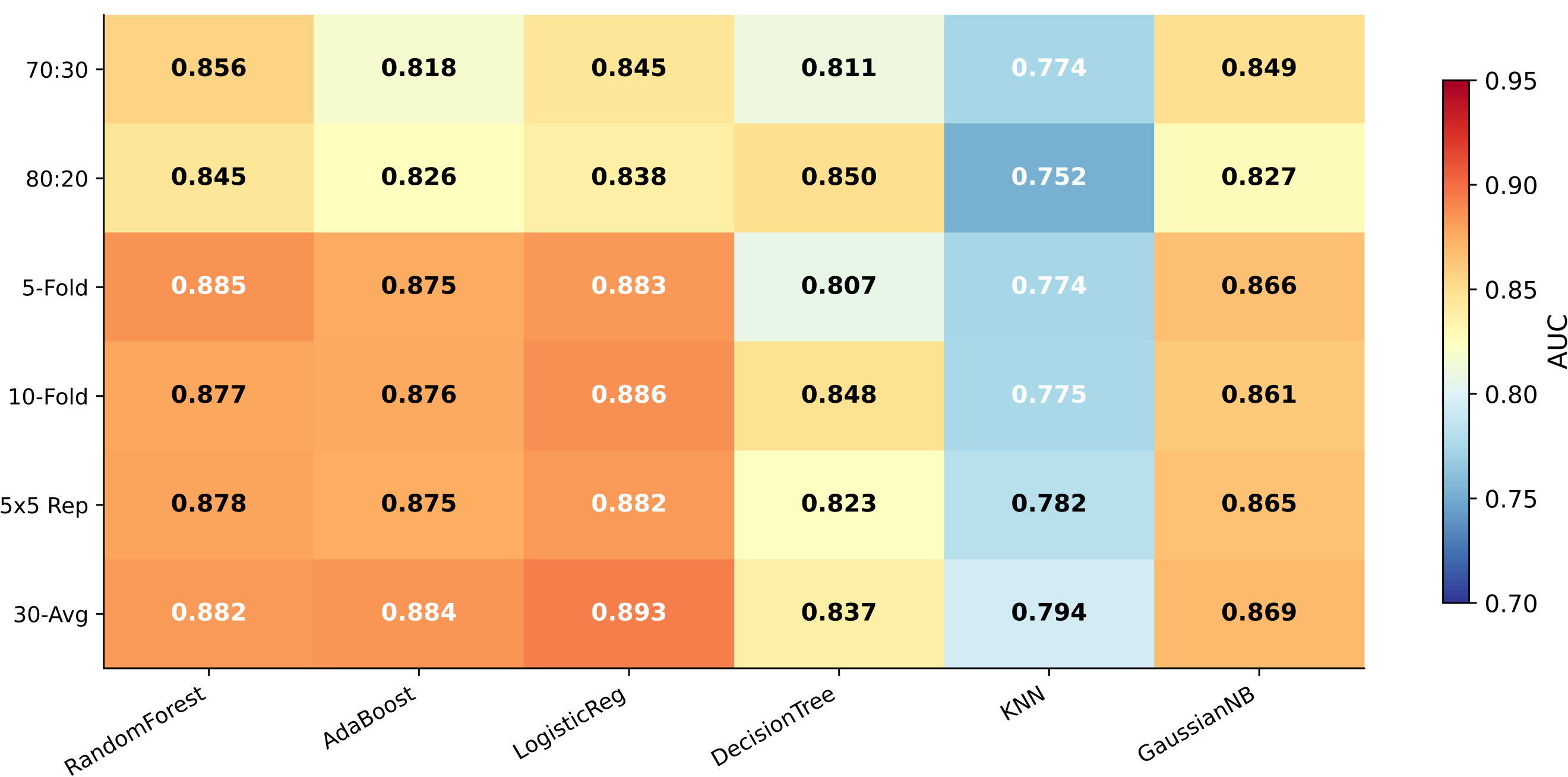


**Figure S11 Heatmap comparing AUC values across six data-splitting strategies.**

AUC values for six ML models evaluated under six different data-splitting strategies: the original 70:30 holdout split, an 80:20 holdout split, 5-fold and 10-fold stratified cross-validation, 5×5 repeated stratified cross-validation, and averaged results from 30 random 70:30 splits. RandomForest maintained consistently competitive AUC values (range: 0.845–0.885) across all strategies, supporting its selection as the optimal model for clinical deployment. Warmer colors (red) indicate higher AUC values; cooler colors (blue) indicate lower AUC values.


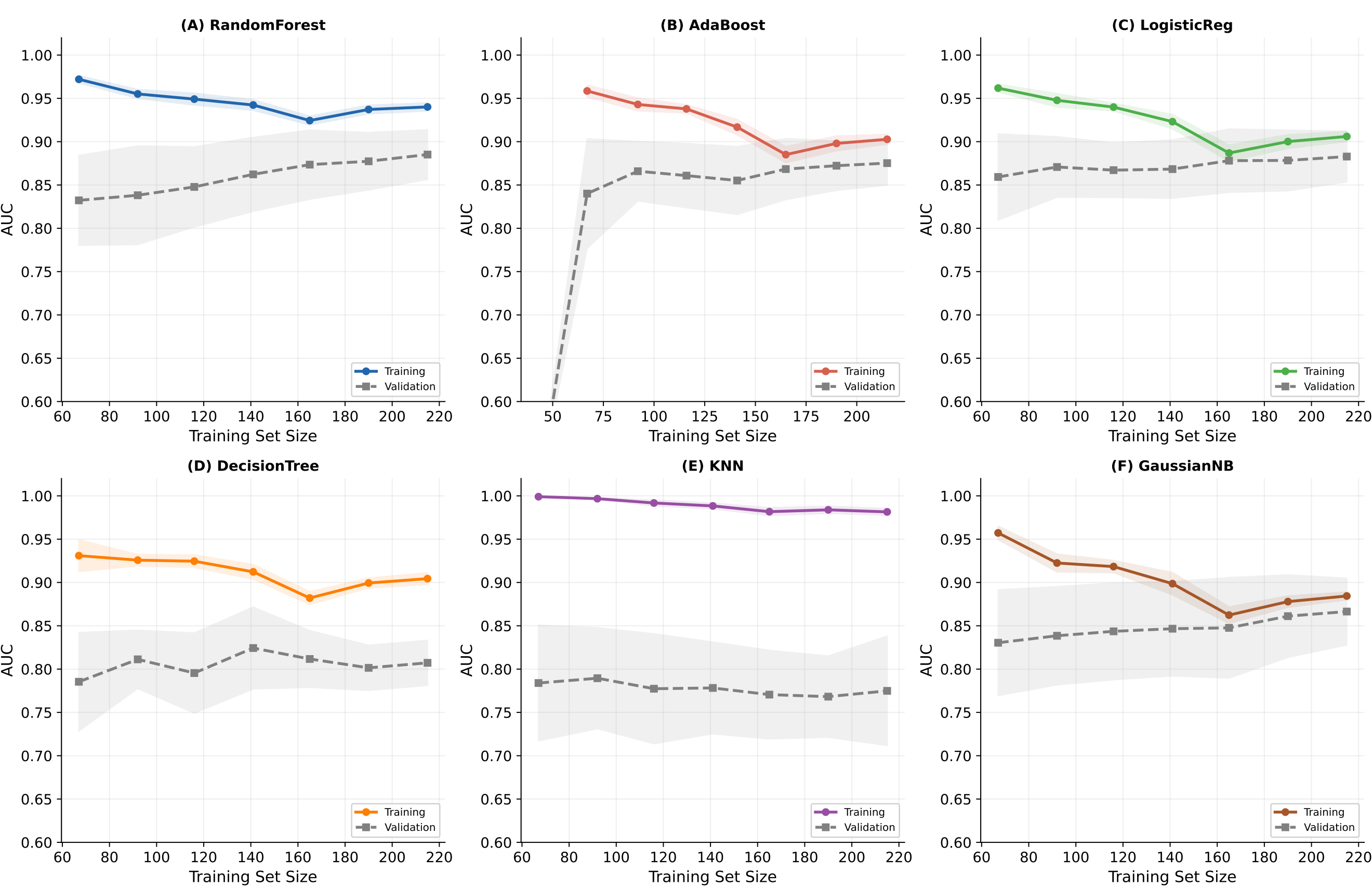


**Figure S12 Learning curves for six ML models.** Learning curves showing the relationship between training set size and model performance (AUC) for each of the six ML algorithms. Shaded regions represent ±1 standard deviation across 5-fold cross-validation folds. (A) RandomForest, (B) AdaBoost, (C) LogisticReg, (D) DecisionTree, (E) KNN, (F) GaussianNB. RandomForest demonstrated convergence of training and validation curves with increasing sample size, indicating good generalization without severe overfitting. KNN showed the largest gap between training and validation curves, suggesting greater susceptibility to overfitting.


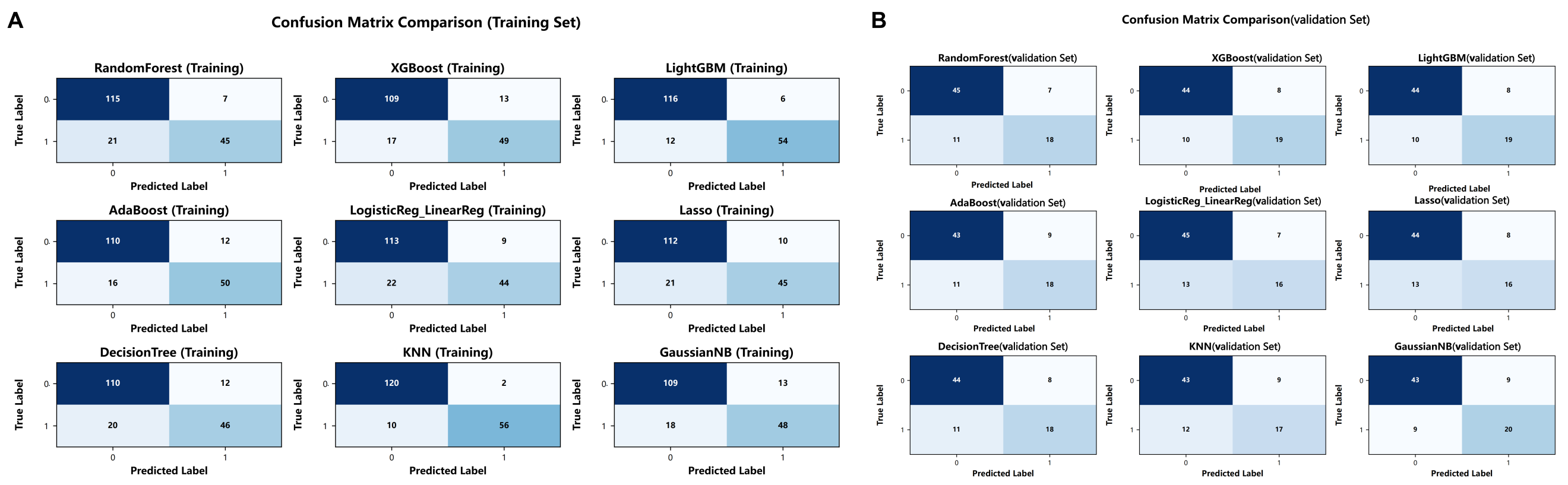


**Figure S13 Comparison of confusion matrices for nine machine learning models in the training and validation cohorts.** (A) Training cohort; (B) validation cohort. The x-axis represents the predicted label, and the y-axis represents the true label. The values in each cell denote the numbers of true negatives, false positives, false negatives, and true positives, respectively.


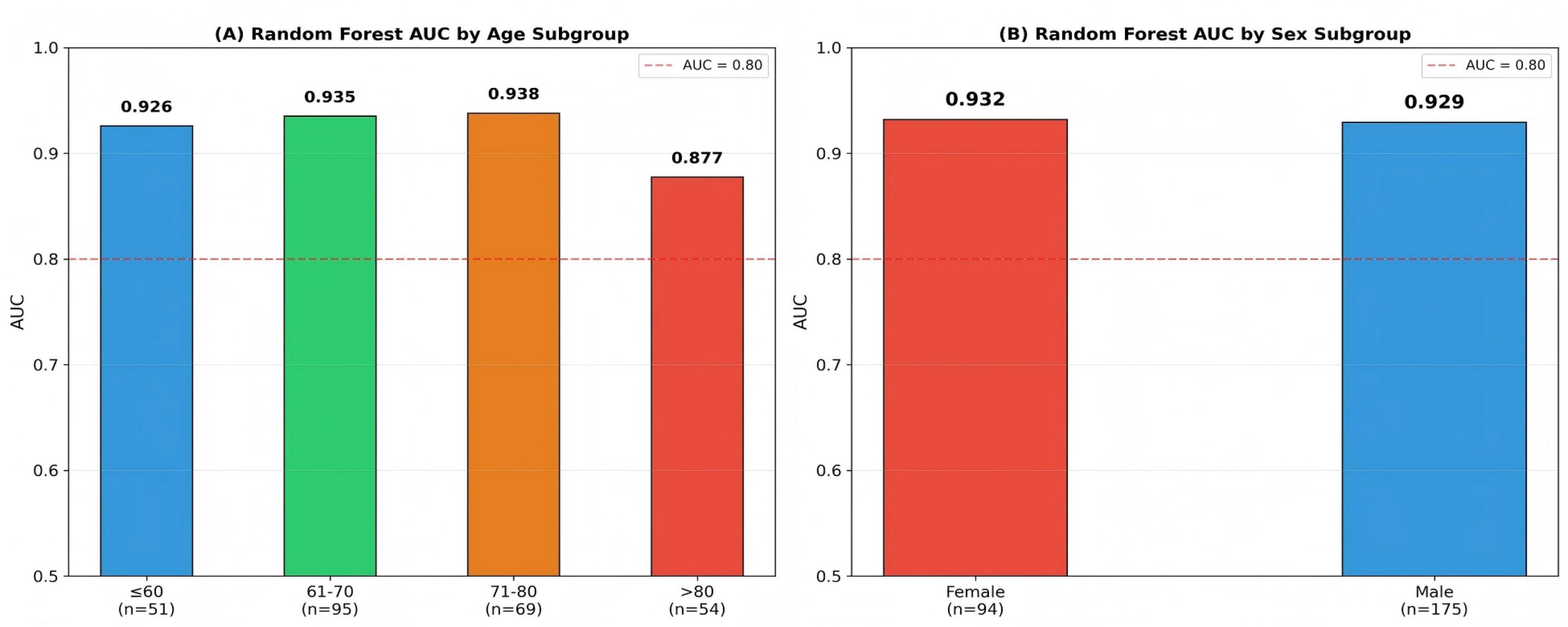


**Figure S14 Subgroup analysis of Random Forest performance.** (A) AUC by age subgroup. (B) AUC by sex subgroup. The model maintained consistent performance across all demographic subgroups. Red dashed line indicates AUC = 0.80 threshold.


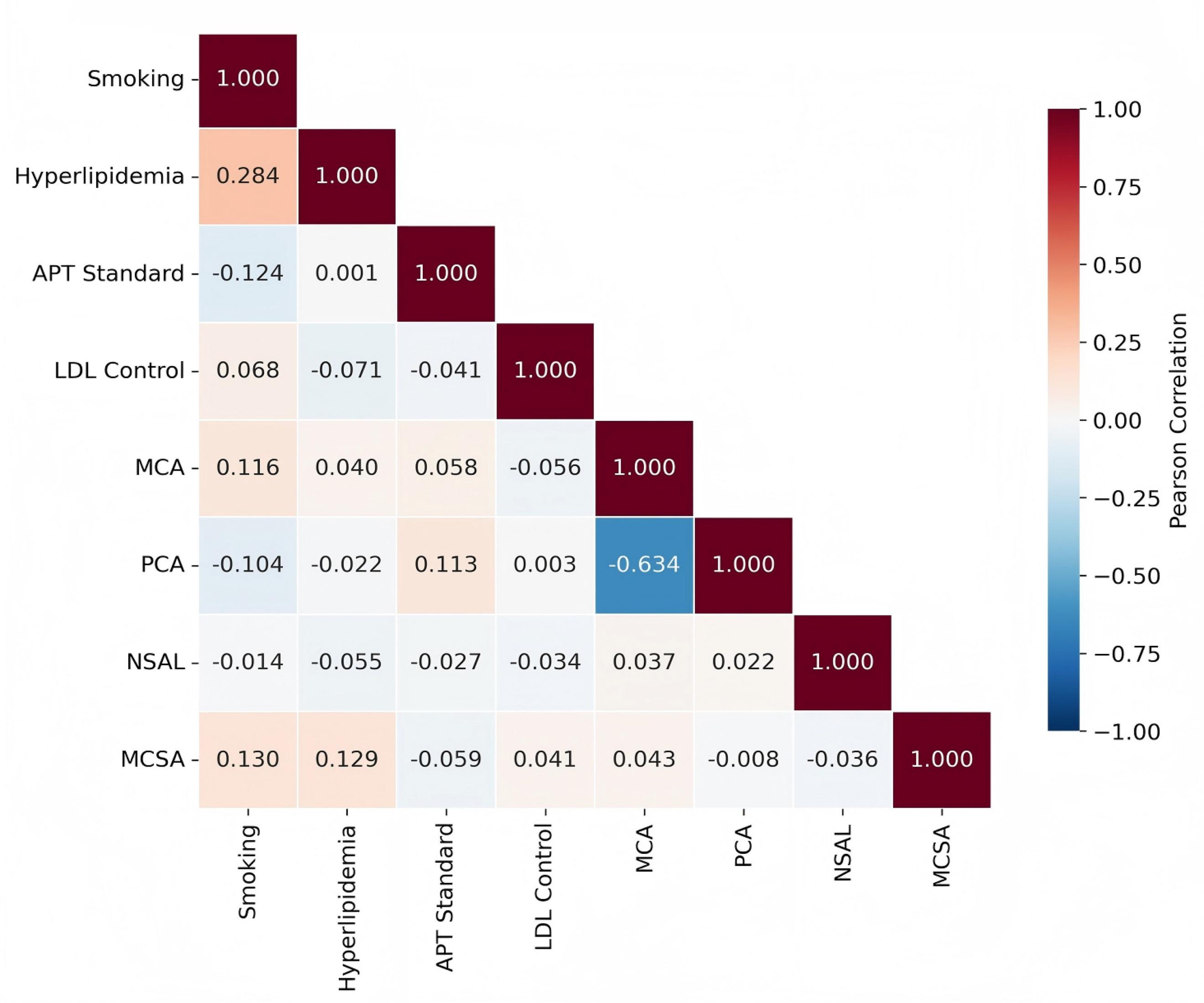


**Figure S15 Pearson correlation matrix of eight selected predictors.** All pairwise |r| < 0.7, confirming absence of severe multicollinearity, consistent with VIF analysis (all VIF < 2.0; Table S5). Color intensity indicates correlation strength (red = positive, blue = negative).

**Figure S16 SHAP dependence plots.** Each plot shows the impact of a feature on the model output, analyzing how different feature values influence the model's prediction. Panels A to H display the impact of smoking history, middle cerebral artery (MCA), posterior cerebral artery (PCA), standardized antiplatelet therapy (APT Standard), maximum cross-sectional area of stroke (MCSA), LDL control, stroke layers (NSAL), and hyperlipidemia (HLD) on the model output.


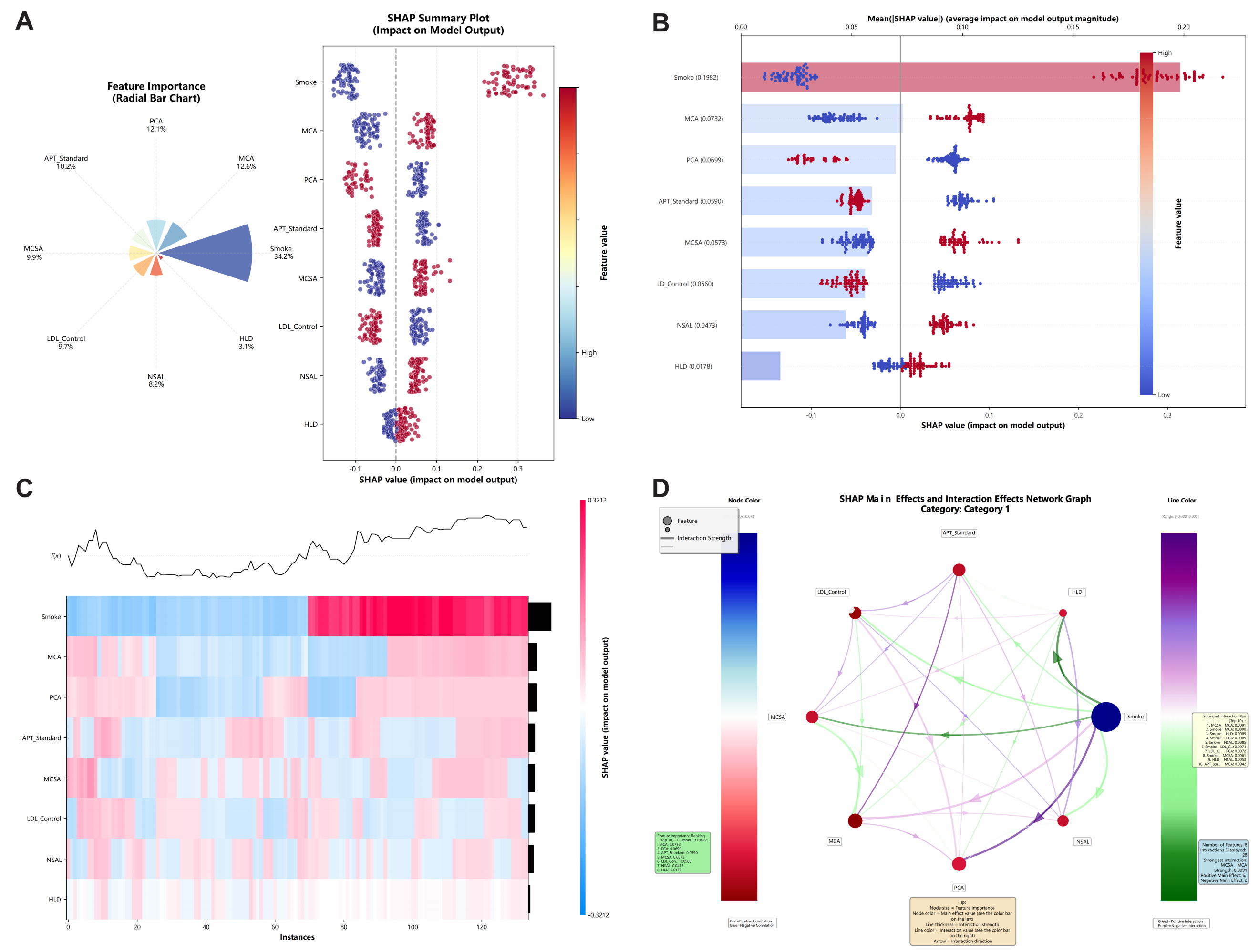


**Figure S17 SHAP-based Visualization Analysis.**
(A) SHAP Summary Plot: The radial bar chart on the left shows feature importance, highlighting the most influential variables. The summary plot on the right demonstrates how the value of each feature impacts the model output, with colors representing the feature values (from low to high). (B) Mean SHAP Value Plot: This bar chart displays the average impact of each feature on the model output. (C) SHAP Heatmap: This heatmap visualizes the SHAP values for different instances, sorted by feature importance. (D) SHAP Effects and Interaction Network Graph: This network graph illustrates the interactions between features and their collective effect on the model output.

**Figure S18 SHAP interaction dependence plots.** (A): Interaction between the maximum cross-sectional area of stroke (MCSA) and the middle cerebral artery (MCA). (B): Interaction between smoking history and MCA. (C): Interaction between smoking history and hyperlipidemia (HLD). (D): Interaction between smoking history and the posterior cerebral artery (PCA). (E): Interaction between smoking history and the number of stroke layers (NSAL).


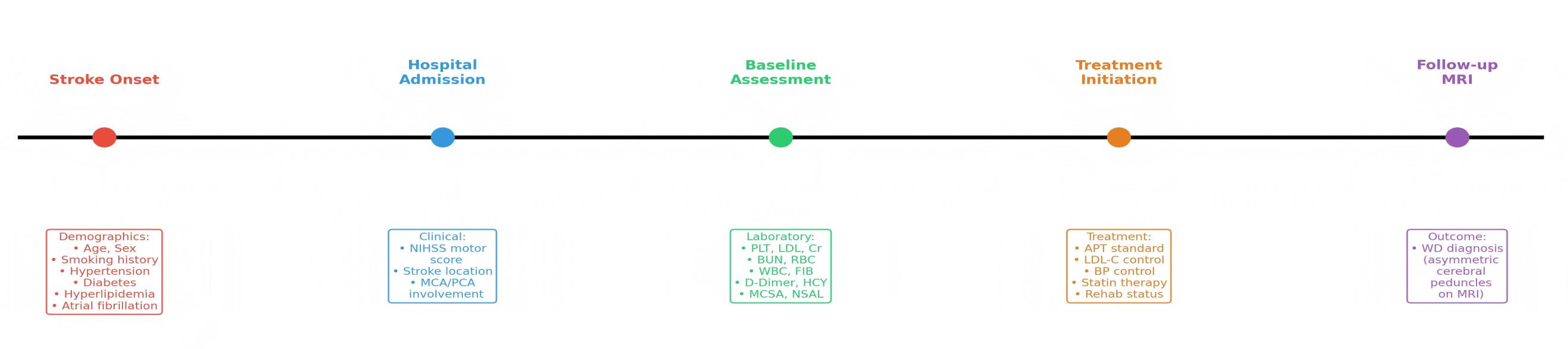


**Figure S19 Timeline of variable measurement relative to stroke onset and WD diagnosis.**

Predictor variables were measured at distinct time points: demographics (pre-existing), clinical and imaging features (acute phase), laboratory parameters (hospitalization), and treatment adequacy (ongoing management). WD was diagnosed on follow-up MRI independently of predictor assessment.


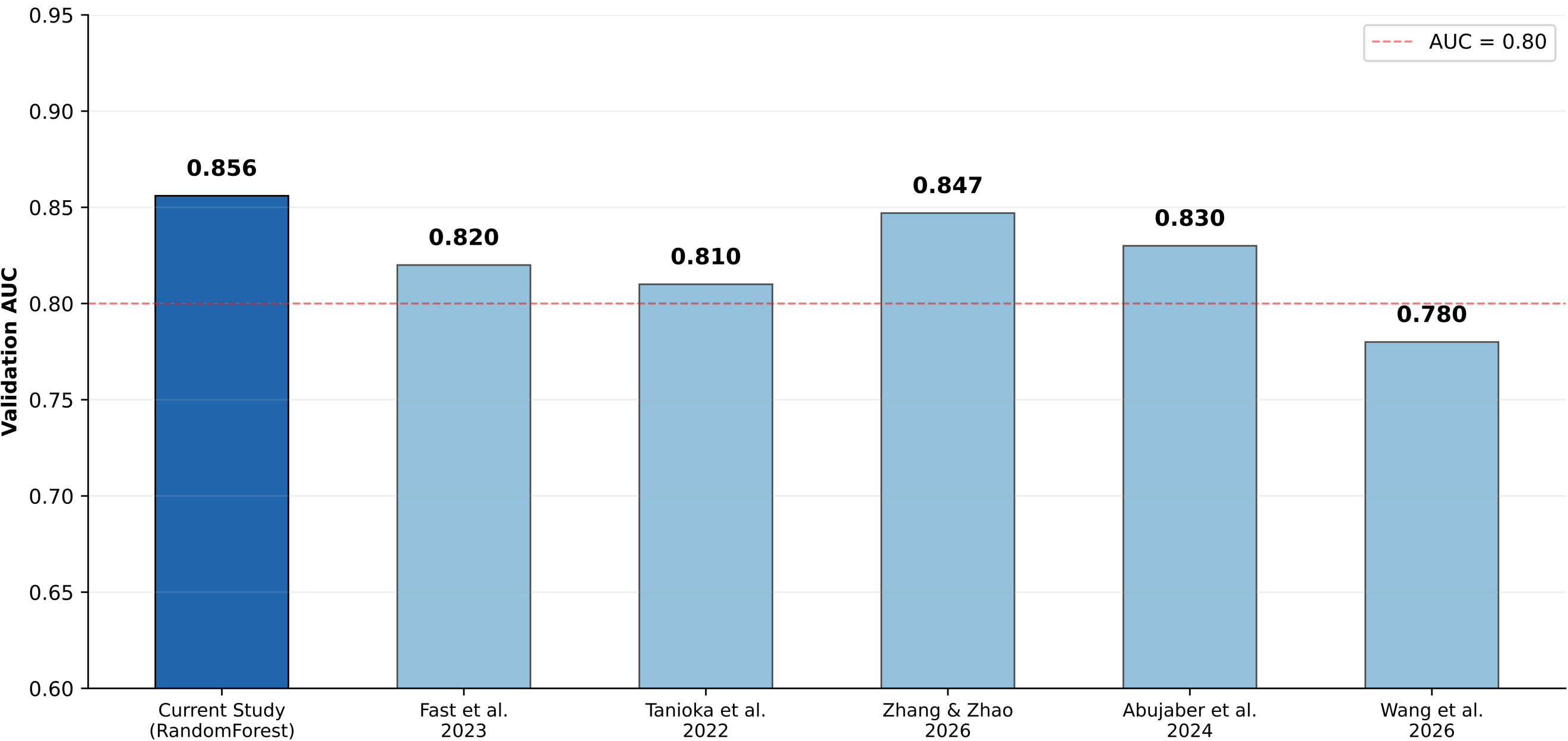


**Figure S20 Comparison of model performance with previous ML studies in stroke research.** Bar chart comparing the validation AUC of our RandomForest model (AUC = 0.856, dark blue) with previously published ML-based prediction studies (light blue) targeting various stroke-related endpoints. Our model achieves comparable or superior performance despite using a more specific endpoint (WD) and fewer features (n = 8). The red dashed line indicates the AUC = 0.80 threshold commonly considered clinically acceptable. Studies compared include:Fast et al. (2023), IS clinical outcome; Tanioka et al. (2022), hematoma expansion; Zhang & Zhao (2026), dysphagia after IS; Abujaber et al. (2024), 90-day stroke prognosis; Wang et al. (2026), IS biomarker signature.


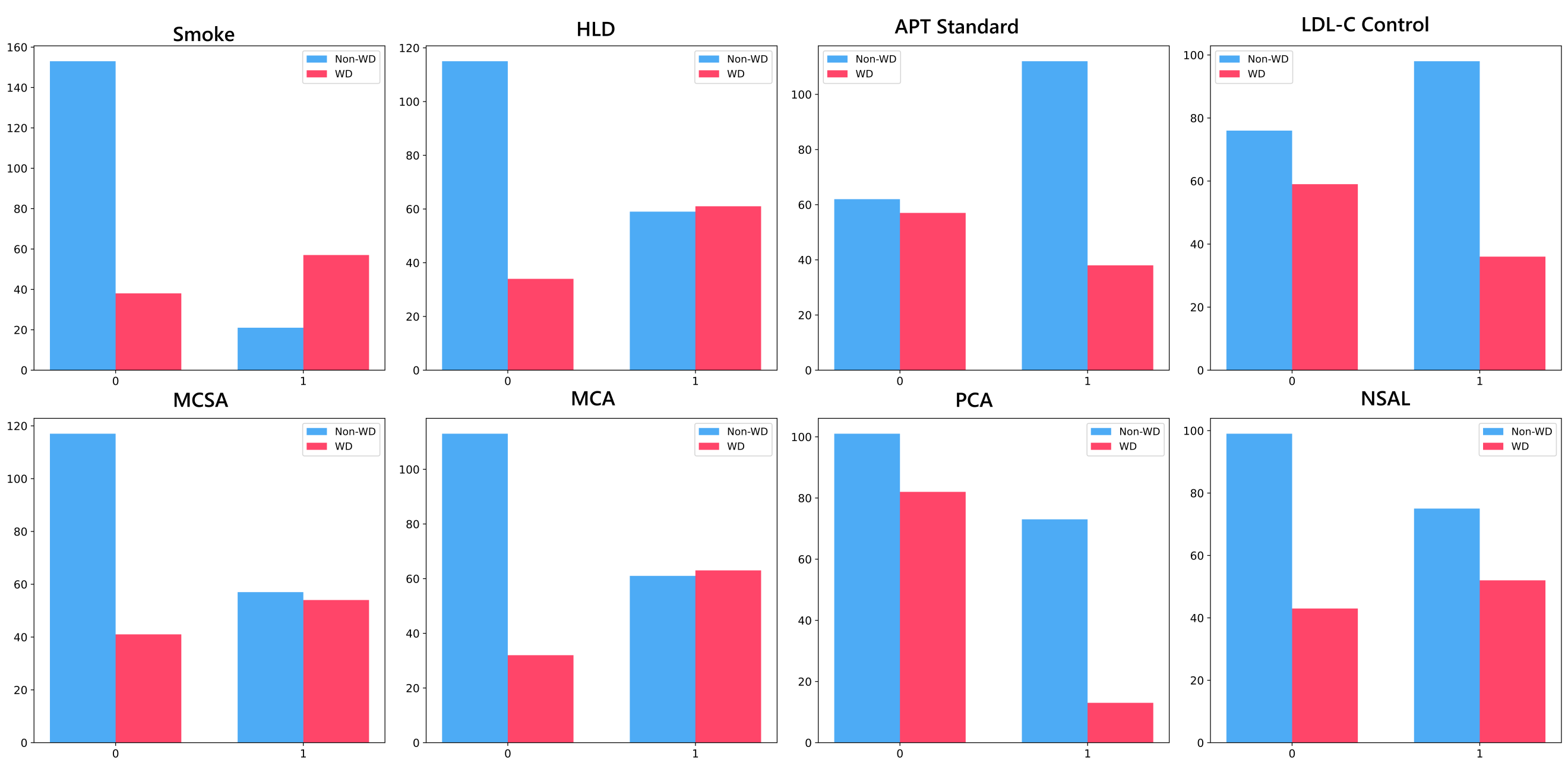


**Figure S21. Distribution of the eight predictive features stratified by Wallerian degeneration (WD) status.** Each panel displays one feature, with blue bars/histogram representing the non-WD group (n = 174) and red bars/histogram representing the WD group (n = 95). For binary variables (Smoke, HLD, APT_Standard, LDL-C Control, MCA, PCA), grouped bar charts show the frequency of each category (0 = absent, 1 = present). For ordinal/continuous variables (MCSA, NSAL), density histograms illustrate the distribution patterns. Smoking history (Smoke) shows the most pronounced separation between WD and non-WD groups, with 60% of WD patients being smokers compared to only 12% of non-WD patients. MCA involvement is present in 66.3% of WD patients vs. 35.1% of non-WD patients. These distributions visually confirm the discriminative value of the selected features. Abbreviations: WD, Wallerian degeneration; Smoke, smoking history; HLD, hyperlipidemia; APT_Standard, standardized antiplatelet therapy; LDL-C Control, LDL cholesterol control; MCA, middle cerebral artery; PCA, posterior cerebral artery; MCSA, maximum cross-sectional area of the stroke; NSAL, number of stroke-affected layers.


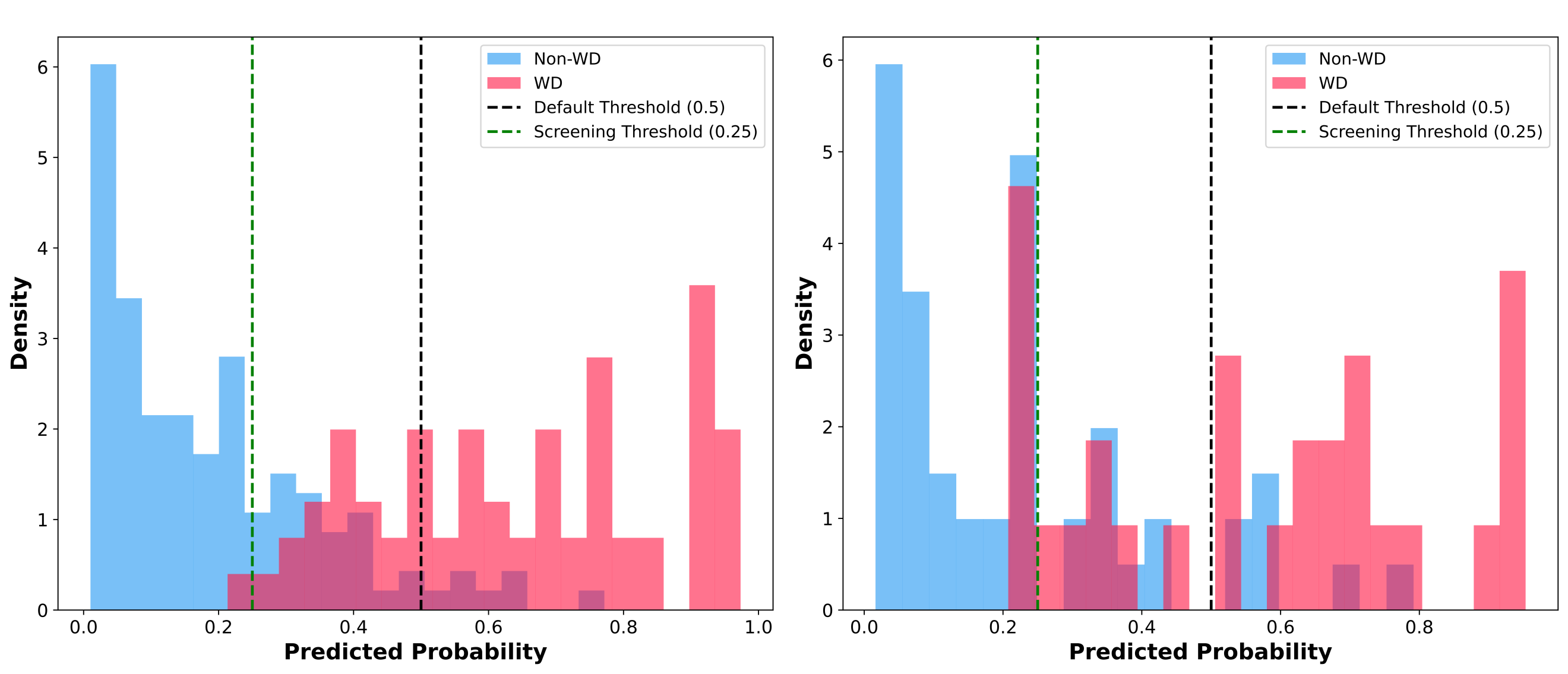


**Figure S22. Distribution of Random Forest predicted probabilities stratified by actual WD status in the training cohort (left) and validation cohort (right).** The x-axis represents the predicted probability of WD (range 0–1), and the y-axis represents the density of samples. Blue histograms represent non-WD patients, and red histograms represent WD patients. The vertical black dashed line indicates the default classification threshold (0.50), and the vertical green dashed line indicates the recommended screening threshold (0.25). Good model discrimination is evidenced by the clear separation between the two distributions in both cohorts. At a threshold of 0.25, the sensitivity reaches 86.2% with an NPV of 90.0%, supporting its use as a screening tool. In the validation cohort, modest overlap between distributions is observed in the 0.2–0.5 range, which is expected given the moderate sample size and reflects the need for clinical judgment in borderline cases. Abbreviations: WD, Wallerian degeneration; RF, Random Forest; NPV, negative predictive value.


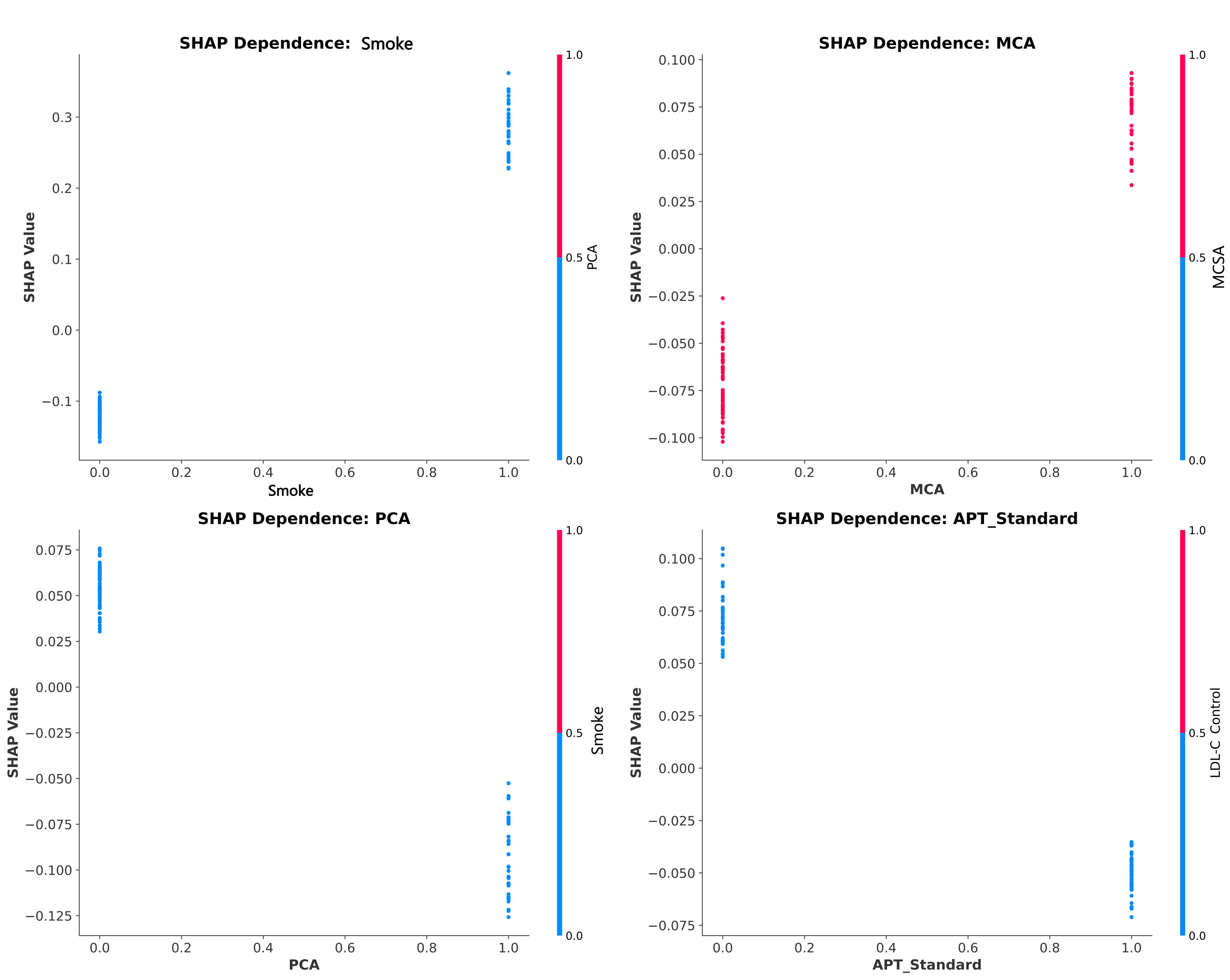


**Figure S23. SHAP dependence plots for the four most influential predictive variables in the Random Forest model: smoking history (Smoke), middle cerebral artery involvement (MCA), posterior cerebral artery involvement (PCA), and standardized antiplatelet therapy (APT_Standard).** In each panel, the x-axis represents the feature value (0 = absent, 1 = present), the y-axis represents the SHAP value (contribution to model output), and the color indicates the value of the most strongly interacting feature (determined automatically by the SHAP algorithm). These plots reveal both the main effects and interaction effects of each feature. For Smoke, the SHAP value transitions from approximately -0.10 (non-smoker) to +0.15–0.30 (smoker), confirming its dominant role as a risk factor. The color gradient reveals interactions with MCA and PCA. For MCA, the transition from non-involvement to involvement increases the SHAP value, with color indicating interaction with smoking status. Abbreviations: SHAP, Shapley Additive Explanations; Smoke, smoking history; MCA, middle cerebral artery; PCA, posterior cerebral artery; APT_Standard, standardized antiplatelet therapy.


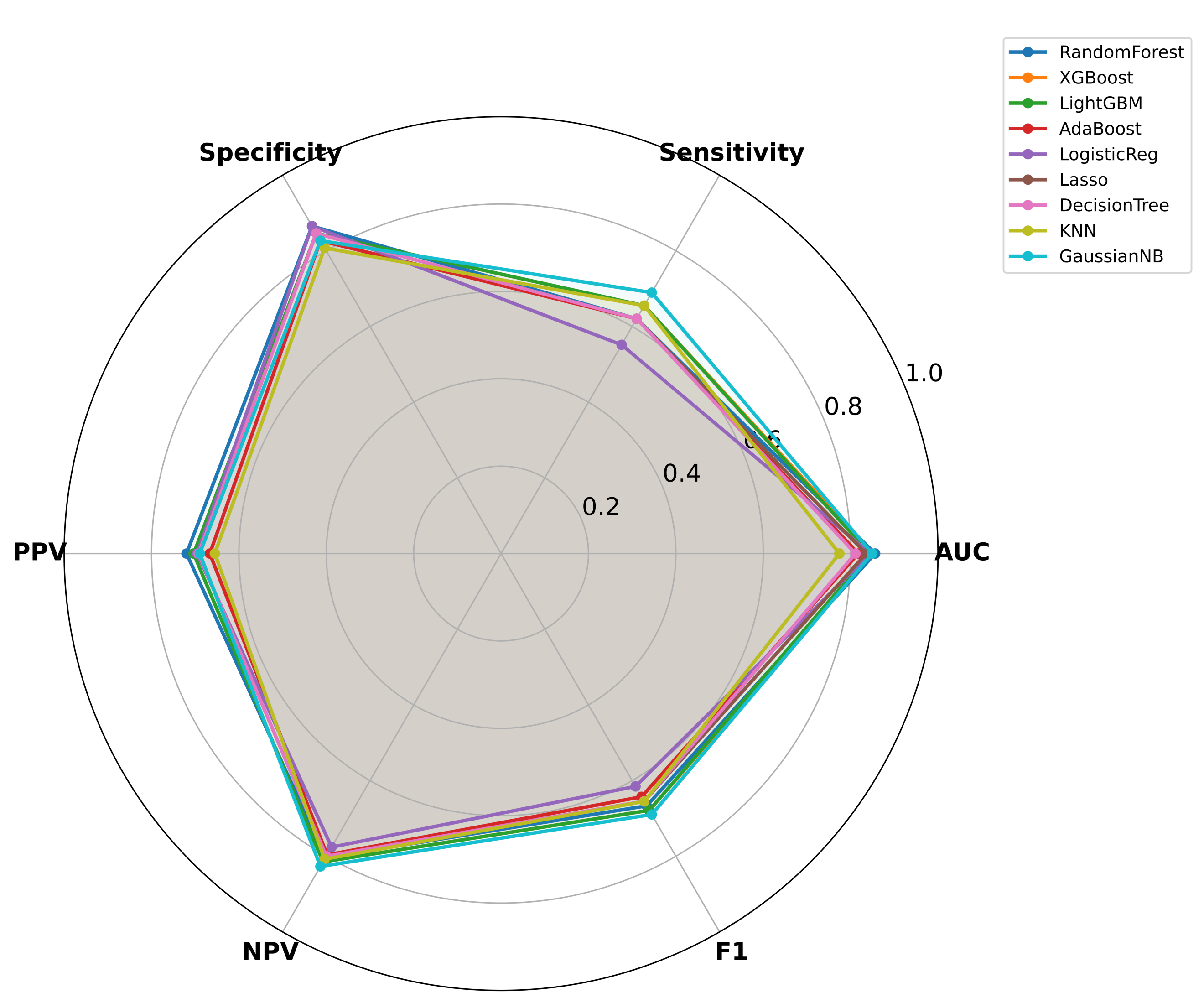


**Figure S24. Radar chart comparing the performance of nine machine learning models across six evaluation metrics in the validation cohort (n = 81).** The six axes represent: AUC (area under the ROC curve), Sensitivity (true positive rate), Specificity (true negative rate), PPV (positive predictive value), NPV (negative predictive value), and F1 Score (harmonic mean of precision and recall). Each colored polygon represents one model, with the area enclosed reflecting overall performance. Models covering larger areas demonstrate more balanced performance across all metrics. Random Forest (blue) shows the most balanced profile with strong specificity (86.5%) and PPV (72.0%), while GaussianNB demonstrates higher sensitivity (69.0%) at the cost of lower specificity. KNN shows the smallest coverage area, consistent with its lowest validation AUC (0.776). This visualization enables clinicians to identify models that best match their priority metrics (e.g., high sensitivity for screening vs. high specificity for confirmation). Abbreviations: AUC, area under the curve; PPV, positive predictive value; NPV, negative predictive value; F1, F1 score; RF, Random Forest; KNN, K-nearest neighbors; NB, Gaussian Naïve Bayes.


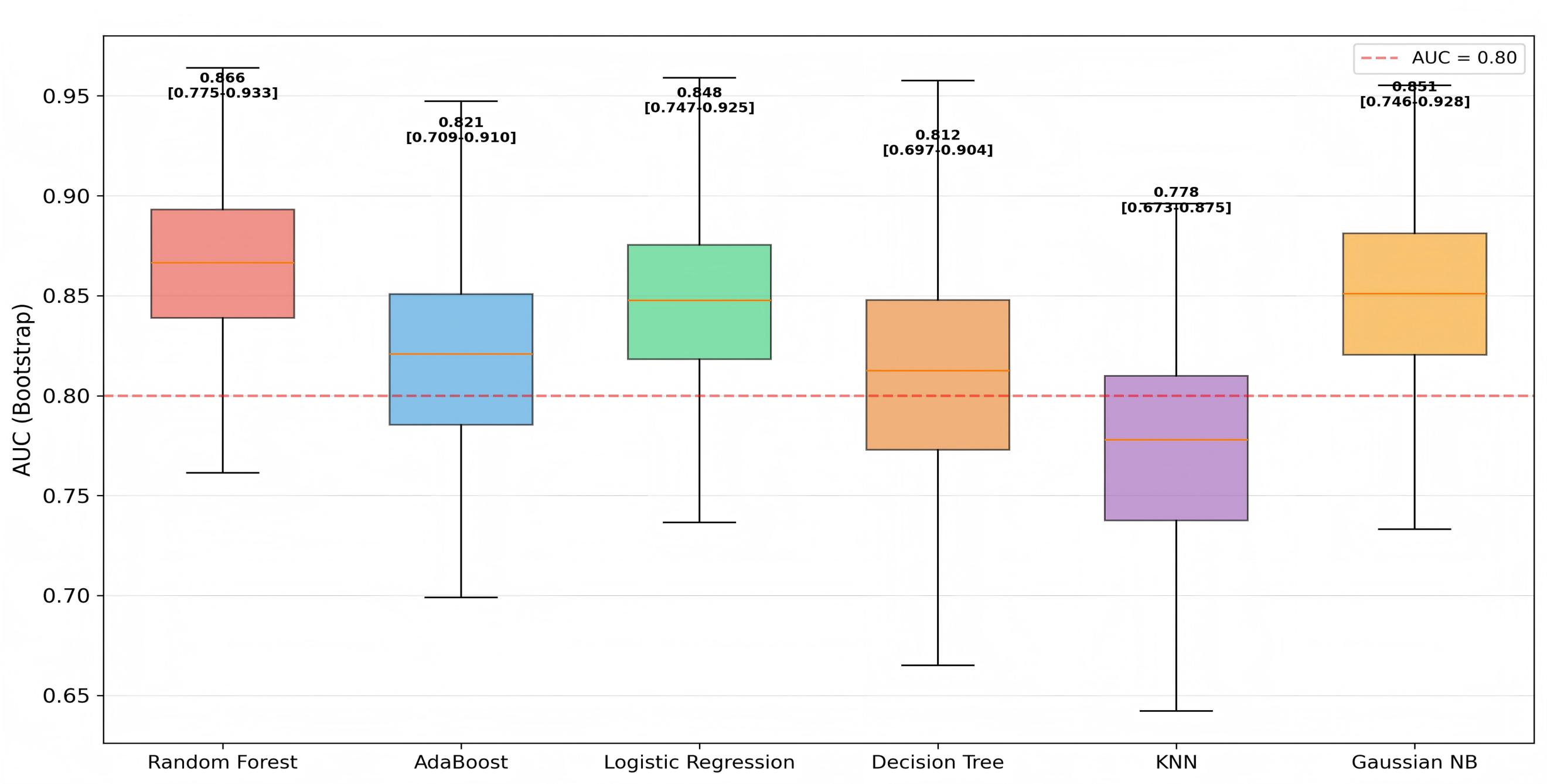


**Figure S25 Bootstrap distribution of validation AUC (500 iterations).**

Box plots showing AUC distributions from 500 bootstrap resamples for six ML models. Median AUC and 95% CI [2.5th–97.5th percentile] are annotated. Random Forest: median = 0.859 (95% CI: 0.753–0.944). Red dashed line indicates AUC = 0.80.


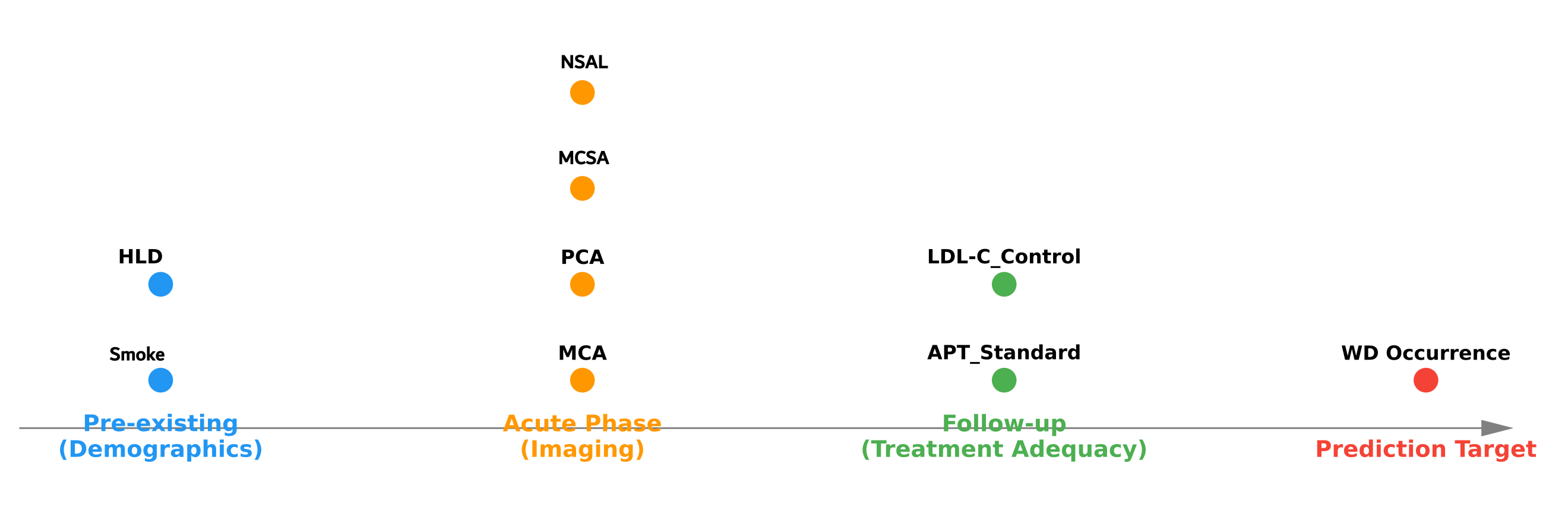


**Figure S26. Schematic illustration of the variable measurement timeline in relation to the clinical pathway for patients with ischemic stroke (IS).** This figure clarifies the temporal framework of the eight predictor variables used in the Random Forest model, which is designed for risk assessment during follow-up rather than acute admission prediction. Variables are categorized into three temporal groups: (1) Pre-existing demographic factors (blue: smoking history [Smoke] and hyperlipidemia [HLD]), which represent stable baseline characteristics present before the index stroke; (2) Acute-phase imaging features (orange: MCA involvement, PCA involvement, maximum cross-sectional area [MCSA], and number of stroke-affected layers [NSAL]), which are measured from cranial MRI during the acute phase of the stroke; and (3) Follow-up treatment adequacy indicators (green: standardized antiplatelet therapy [APT_Standard] and LDL-C control [LDL-C_Control]), which reflect the quality of ongoing secondary prevention management assessed during follow-up visits. The prediction target (red: WD occurrence) is the binary outcome evaluated at the follow-up time point. This temporal framework supports the clinical positioning of the model as a follow-up monitoring tool. Abbreviations: WD, Wallerian degeneration; IS, ischemic stroke; MCA, middle cerebral artery; PCA, posterior cerebral artery; APT, antiplatelet therapy; LDL-C, low-density lipoprotein cholesterol; MRI, magnetic resonance imaging.
