# Supplementary material for: Establishment of a machine learning prediction model for Wallerian degeneration after ischemic stroke
Source: Front Med (Lausanne). 2026 Jun 30;13:1841202. doi: 10.3389/fmed.2026.1841202 (PMC13365044; doi:10.3389/fmed.2026.1841202)
Supplement: Supplementary file 2 [file Supplementary_file_2.DOCX]

| **Category** | **N** | **Percentage** |
| --- | --- | --- |
| Total IS patient records screened | 308 | 100.00% |
| Excluded - Total | 39 | 12.70% |
| Newly diagnosed IS | 10 | 3.20% |
| WD caused by non-ischemic Stroke | 15 | 4.90% |
| Incomplete mRS/MMSE/MRI | 14 | 4.50% |
| Included in final analysis | 269 | 87.30% |
| Training cohort (70%) | 188 | 69.90% |
| WD group | 66 | 35.10% |
| Non-WD group | 122 | 64.90% |
| Validation cohort (30%) | 81 | 30.10% |
| WD group | 29 | 35.80% |
| Non-WD group | 52 | 64.20% |

**Table S1 Summary of Participant Enrollment Inclusion and Exclusion.**

**Table S2 Baseline characteristics of the training and validation cohorts in patients with IS.**

|  | **ALL (N=269)** | **Test (N=81)** | **Train (N=188)** | **p-value** |
| --- | --- | --- | --- | --- |
| **Gender, n (%)** |  |  |  | 0.045 |
| Female | 94 (34.94%) | 36 (44.44%) | 58 (30.85%) |  |
| Male | 175 (65.06%) | 45 (55.56%) | 130 (69.15%) |  |
| **Age (year)** | 70.00  [65.00;77.00] | 70.00  [65.00;78.00] | 70.00  [64.00;77.00] | 0.609 |
| **Smoke, n (%)** |  |  |  | 0.144 |
| No | 191 (71.00%) | 63 (77.78%) | 128 (68.09%) |  |
| Yes | 78 (29.00%) | 18 (22.22%) | 60 (31.91%) |  |
| **Hypertension, n (%)** |  |  |  | 0.551 |
| No | 104 (38.66%) | 34 (41.98%) | 70 (37.23%) |  |
| Yes | 165 (61.34%) | 47 (58.02%) | 118 (62.77%) |  |
| **Hyperlipidemia, n (%)** |  |  |  | 0.132 |
| No | 149 (55.39%) | 51 (62.96%) | 98 (52.13%) |  |
| Yes | 120 (44.61%) | 30 (37.04%) | 90 (47.87%) |  |
| **Diabetes Mellitus, n (%)** |  |  |  | 1 |
| No | 174 (64.68%) | 52 (64.20%) | 122 (64.89%) |  |
| Yes | 95 (35.32%) | 29 (35.80%) | 66 (35.11%) |  |
| **Atrial Fibrillation, n (%)** |  |  |  | 0.82 |
| No | 236 (87.73%) | 70 (86.42%) | 166 (88.30%) |  |
| Yes | 33 (12.27%) | 11 (13.58%) | 22 (11.70%) |  |
| **CTX, n (%)** |  |  |  | 0.945 |
| No | 142 (52.79%) | 42 (51.85%) | 100 (53.19%) |  |
| Yes | 127 (47.21%) | 39 (48.15%) | 88 (46.81%) |  |
| **CSO, n (%)** |  |  |  | 0.433 |
| No | 262 (97.40%) | 78 (96.30%) | 184 (97.87%) |  |
| Yes | 7 (2.60%) | 3 (3.70%) | 4 (2.13%) |  |
| **LV, n (%)** |  |  |  | 0.845 |
| No | 152 (56.51%) | 47 (58.02%) | 105 (55.85%) |  |
| Yes | 117 (43.49%) | 34 (41.98%) | 83 (44.15%) |  |
| **IC, n (%)** |  |  |  | 0.927 |
| No | 230 (85.50%) | 70 (86.42%) | 160 (85.11%) |  |
| Yes | 39 (14.50%) | 11 (13.58%) | 28 (14.89%) |  |
| **BS, n (%)** |  |  |  | 0.293 |
| No | 228 (84.76%) | 72 (88.89%) | 156 (82.98%) |  |
| Yes | 41 (15.24%) | 9 (11.11%) | 32 (17.02%) |  |
| **MCSA, n (%)** |  |  |  | 1 |
| No | 158 (58.74%) | 48 (59.26%) | 110 (58.51%) |  |
| Yes | 111 (41.26%) | 33 (40.74%) | 78 (41.49%) |  |
| **MCA, n (%)** |  |  |  | 0.399 |
| No | 145 (53.90%) | 40 (49.38%) | 105 (55.85%) |  |
| Yes | 124 (46.10%) | 41 (50.62%) | 83 (44.15%) |  |
| **ACA, n (%)** |  |  |  | 0.904 |
| No | 136 (50.56%) | 40 (49.38%) | 96 (51.06%) |  |
| Yes | 133 (49.44%) | 41 (50.62%) | 92 (48.94%) |  |
| **PCA, n (%)** |  |  |  | 0.21 |
| No | 183 (68.03%) | 60 (74.07%) | 123 (65.43%) |  |
| Yes | 86 (31.97%) | 21 (25.93%) | 65 (34.57%) |  |
| **NSAL > 2, n (%)** |  |  |  | 0.843 |
| No | 142 (52.79%) | 44 (54.32%) | 98 (52.13%) |  |
| Yes | 127 (47.21%) | 37 (45.68%) | 90 (47.87%) |  |
| **NIHSS limbs** | 1.00  [0.00;2.00] | 1.00  [0.00;2.00] | 1.00  [0.00;2.00] | 0.355 |
| **APT Standard, n (%)** |  |  |  | 0.721 |
| No | 119 (44.24%) | 34 (41.98%) | 85 (45.21%) |  |
| Yes | 150 (55.76%) | 47 (58.02%) | 103 (54.79%) |  |
| **BP Control, n (%)** |  |  |  | 0.989 |
| No | 131 (48.70%) | 40 (49.38%) | 91 (48.40%) |  |
| Yes | 138 (51.30%) | 41 (50.62%) | 97 (51.60%) |  |
| **LDL Control, n (%)** |  |  |  | 0.968 |
| No | 135 (50.19%) | 40 (49.38%) | 95 (50.53%) |  |
| Yes | 134 (49.81%) | 41 (50.62%) | 93 (49.47%) |  |
| **SNR, n (%)** |  |  |  | 0.068 |
| No | 124 (46.10%) | 30 (37.04%) | 94 (50.00%) |  |
| Yes | 145 (53.90%) | 51 (62.96%) | 94 (50.00%) |  |
| **Cr (mg/dl)** | 77.00  [66.00;80.00] | 77.00  [67.00;80.00] | 77.00  [66.00;80.00] | 0.798 |
| **BUN (mg/dl)** | 5.00  [4.00;5.00] | 4.30  [4.00;5.00] | 5.00  [4.00;5.00] | 0.383 |
| **RBC (10^9^ /L)** | 5.00  [4.50;5.00] | 5.00  [4.50;5.00] | 5.00  [4.50;5.00] | 0.568 |
| **PLT (10^9^ /L)** | 211.00  [165.00;258.00] | 217.00 [152.00;254.00] | 210.00  [169.00;258.00] | 0.592 |
| **WBC (10^9^ /L)** | 6.50  [4.70;8.20] | 6.80  [4.90;8.30] | 6.40  [4.60;8.20] | 0.231 |
| **FIB (g/L)** | 3.00  [2.50;3.60] | 3.00  [2.50;3.60] | 3.00  [2.50;3.60] | 0.725 |
| **D-Dimer (μg/L)** | 0.05  [0.03;0.07] | 0.05  [0.02;0.07] | 0.05  [0.03;0.08] | 0.204 |
| **HCY (μmol/L)** | 9.50  [6.70;12.30] | 9.90  [6.90;12.50] | 9.35  [6.65;12.22] | 0.721 |

Abbreviations: CTX, Cerebral Cortex; CSO, Centrum Semiovale; LV, Lateral Ventricle; IC, Internal Capsule; BS, Brainstem; MCA, Middle Cerebral Artery; ACA, Anterior Cerebral Artery; PCA, Posterior Cerebral Artery; MCSA, Maximum Cross-Sectional Area; NSAL, Number of Stroke-Affected Layers; NIHSS limbs, National Institutes of Health Stroke Scale - Limbs subscore; APT Standard, Antiplatelet Therapy-Standard; BP Control,Blood Pressure Control; LDL Control, Low-Density Lipoprotein Control; SNR, Standardized Neurological Rehabilitation; Cr, Creatinine; BUN, Blood Urea Nitrogen; RBC, Red Blood Cell (count); PLT, Platelet (count); WBC, White Blood Cell (count); FIB, Fibrinogen; HCY, Homocysteine.

**Table S3 Comparison of baseline clinical and laboratory characteristics between IS patients with and without WD showed no statistically significant differences.**

|  | **Total (N=269)** | **No (N=174)** | **Yes (N=95)** | **p-value** |
| --- | --- | --- | --- | --- |
| **Gender, n (%)** |  |  |  | 0.323 |
| Female | 94 (34.94%) | 65 (37.36%) | 29 (30.53%) |  |
| Male | 175 (65.06%) | 109 (62.64%) | 66 (69.47%) |  |
| **Age (year)** | 70 | 70 | 69 | 0.432 |
|  | [65.00;77.00] | [64.00;78.75] | [65.00;75.00] |  |
| **Hypertension, n (%)** |  |  |  | 0.559 |
| No | 104 (38.66%) | 70 (40.23%) | 34 (35.79%) |  |
| Yes | 165 (61.34%) | 104 (59.77%) | 61 (64.21%) |  |
| **Diabetes Mellitus, n (%)** |  |  |  | 0.017 |
| No | 174 (64.68%) | 122 (70.11%) | 52 (54.74%) |  |
| Yes | 95 (35.32%) | 52 (29.89%) | 43 (45.26%) |  |
| **Atrial Fibrillation, n (%)** |  |  |  | 1 |
| No | 236 (87.73%) | 153 (87.93%) | 83 (87.37%) |  |
| Yes | 33 (12.27%) | 21 (12.07%) | 12 (12.63%) |  |
| **CTX, n (%)** |  |  |  | 0.73 |
| No | 142 (52.79%) | 90 (51.72%) | 52 (54.74%) |  |
| Yes | 127 (47.21%) | 84 (48.28%) | 43 (45.26%) |  |
| **CSO, n (%)** |  |  |  | 0.7 |
| No | 262 (97.40%) | 170 (97.70%) | 92 (96.84%) |  |
| Yes | 7 (2.60%) | 4 (2.30%) | 3 (3.16%) |  |
| **LV, n (%)** |  |  |  | 0.833 |
| No | 152 (56.51%) | 97 (55.75%) | 55 (57.89%) |  |
| Yes | 117 (43.49%) | 77 (44.25%) | 40 (42.11%) |  |
| **IC, n (%)** |  |  |  | 0.323 |
| No | 230 (85.50%) | 152 (87.36%) | 78 (82.11%) |  |
| Yes | 39 (14.50%) | 22 (12.64%) | 17 (17.89%) |  |
| **BS, n (%)** |  |  |  | 0.728 |
| No | 228 (84.76%) | 146 (83.91%) | 82 (86.32%) |  |
| Yes | 41 (15.24%) | 28 (16.09%) | 13 (13.68%) |  |
| **ACA, n (%)** |  |  |  | 0.529 |
| No | 136 (50.56%) | 85 (48.85%) | 51 (53.68%) |  |
| Yes | 133 (49.44%) | 89 (51.15%) | 44 (46.32%) |  |
| **NSAL > 2, n (%)** |  |  |  | 0.089 |
| No | 142 (52.79%) | 99 (56.90%) | 43 (45.26%) |  |
| Yes | 127 (47.21%) | 75 (43.10%) | 52 (54.74%) |  |
| **NIHSS limbs** | 1 | 1 | 1 | 0.096 |
|  | [0.00;2.00] | [1.00;2.00] | [0.00;3.00] |  |
| **BP Control, n (%)** |  |  |  | 0.111 |
| No | 131 (48.70%) | 78 (44.83%) | 53 (55.79%) |  |
| Yes | 138 (51.30%) | 96 (55.17%) | 42 (44.21%) |  |
| **LDL Control, n (%)** |  |  |  | 0.006 |
| No | 135 (50.19%) | 76 (43.68%) | 59 (62.11%) |  |
| Yes | 134 (49.81%) | 98 (56.32%) | 36 (37.89%) |  |
| **SNR, n (%)** |  |  |  | 0.013 |
| No | 124 (46.10%) | 70 (40.23%) | 54 (56.84%) |  |
| Yes | 145 (53.90%) | 104 (59.77%) | 41 (43.16%) |  |
| **Cr (mg/dl)** | 77 | 76 | 77 | 0.604 |
|  | [66.00;80.00] | [66.00;80.00] | [66.50;80.00] |  |
| **BUN (mg/dl)** | 5 | 5 | 4.3 | 0.029 |
|  | [4.00;5.00] | [4.00;5.00] | [4.00;5.00] |  |
| **RBC (109 /L)** | 5 | 5 | 5 | 0.826 |
|  | [4.50;5.00] | [4.50;5.00] | [4.50;5.00] |  |
| **PLT (109 /L)** | 211.00 [165.00;258.00] | 211.00 [166.25;258.00] | 209.00 [161.00;256.00] | 0.749 |
| **WBC (109 /L)** | 6.5 | 6.45 | 6.5 | 0.973 |
|  | [4.70;8.20] | [4.73;8.20] | [4.75;8.25] |  |
| **FIB (g/L)** | 3 | 3 | 3 | 0.992 |
|  | [2.50;3.60] | [2.50;3.60] | [2.50;3.60] |  |
| **D-Dimer (μg/L)** | 0.05 | 0.05 | 0.05 | 0.935 |
|  | [0.03;0.07] | [0.03;0.07] | [0.03;0.07] |  |
| **HCY (μmol/L)** | 9.5 | 9.55 | 9.4 | 0.974 |
|  | [6.70;12.30] | [6.70;12.28] | [6.75;12.25] |  |

Abbreviations: CTX, Cerebral Cortex; CSO, Centrum Semiovale; LV, Lateral Ventricle; IC, Internal Capsule; BS, Brainstem; ACA, Anterior Cerebral Artery; NSAL, Number of Stroke-Affected Layers; NIHSS limbs, National Institutes of Health Stroke Scale - Limbs subscore; BP Control,Blood Pressure Control; LDL Control, Low-Density Lipoprotein Control; SNR, Standardized Neurological Rehabilitation; Cr, Creatinine; BUN, Blood Urea Nitrogen; RBC, Red Blood Cell (count); PLT, Platelet (count); WBC, White Blood Cell (count); FIB, Fibrinogen; HCY, Homocysteine;

**Table S4 Variables selected by LASSO regression with corresponding coefficients at Lambda.1se.**

| **Variable** | **Coefficient (β)** |
| --- | --- |
| Smoke | 1.84478059 |
| Hyperlipidemia | 0.46858223 |
| MCA | 0.33613374 |
| NSAL | 0.01813518 |
| MCSA | 0.0127935 |
| PCA | -0.18175487 |
| LDL Control | -0.33651309 |
| APT Standard | -0.68976836 |

Abbreviations: MCA, Middle Cerebral Artery; NSAL, Number of Stroke-Affected Layers; MCSA, Maximum Cross-Sectional Area; PCA, Posterior Cerebral Artery; LDL Control, Low-Density Lipoprotein Control; APT Standard, Antiplatelet Therapy-Standard;

**Table S5 Variance inflation factor (VIF) values for variables included in the multivariate logistic regression model.**

|  | **VIF** |
| --- | --- |
| Smoke | 1.419501199 |
| Hyperlipidemia | 1.103841881 |
| APT Standard | 1.344105837 |
| LDL Control | 1.240157082 |
| MCSA | 1.105876207 |
| MCA | 1.727279876 |
| PCA | 1.678764558 |
| NSAL | 1.128846294 |

**Table S6 Hyperparameters optimized through Random Search for each ML model.**

| **Model** | **Tuning Method** | **Best Hyperparameters** |
| --- | --- | --- |
| Random Forest | Random Search | {'n_estimators': 300, 'min_samples_split': 10, 'min_samples_leaf': 2, 'max_features': 'log2', 'max_depth': 20} |
| XGBoost | Random Search | {'subsample': 1.0, 'n_estimators': 200, 'min_child_weight': 5, 'max_depth': 5, 'learning_rate': 0.05, 'gamma': 0.1, 'colsample_bytree': 0.8} |
| LightGBM | Random Search | {'subsample': 0.8, 'num_leaves': 100, 'n_estimators': 50, 'min_child_samples': 5, 'max_depth': 7, 'learning_rate': 0.05, 'colsample_bytree': 0.8} |
| AdaBoost | Random Search | {'n_estimators': 100, 'learning_rate': 0.1} |
| Logistic Regression | Random Search | {'C': 1} |
| Decision Tree | Random Search | {'min_samples_split': 20, 'min_samples_leaf': 8, 'max_depth': 7, 'criterion': 'entropy'} |
| KNN | Random Search | {'weights': 'distance', 'n_neighbors': 15, 'metric': 'manhattan'} |

Abbreviations: KNN, K-nearest neighbors; NB, Naïve Bayes.

**Table S7 Comparison of AUC values of eight ML models in the training cohort.**

|  | Random Forest | XGBoost | LightGBM | AdaBoost | Logistic Regression | Decision Tree | KNN | GaussianNB |
| --- | --- | --- | --- | --- | --- | --- | --- | --- |
| Random Forest | 1 | 0.028414 | 0.000815 | 0.002241 | 0.002405 | 0.002923 | 0.000437 | 0.000847 |
| XGBoost | 0.028414 | 1 | 0.000248 | 0.072725 | 0.108509 | 0.066458 | 0.000193 | 0.020877 |
| LightGBM | 0.000815 | 0.000248 | 1 | 0.000136 | 0.000173 | 0.00028 | 0.012234 | 0.00015 |
| AdaBoost | 0.002241 | 0.072725 | 0.000136 | 1 | 0.436439 | 0.497792 | 0.000047 | 0.211663 |
| Logistic Regression | 0.002405 | 0.108509 | 0.000173 | 0.436439 | 1 | 0.343615 | 0.000057 | 0.08261 |
| Decision Tree | 0.002923 | 0.066458 | 0.00028 | 0.497792 | 0.343615 | 1 | 0.000057 | 0.719585 |
| KNN | 0.000437 | 0.000193 | 0.012234 | 0.000047 | 0.000057 | 0.000057 | 1 | 0.000054 |
| GaussianNB | 0.000847 | 0.020877 | 0.00015 | 0.211663 | 0.08261 | 0.719585 | 0.000054 | 1 |

Abbreviations: KNN, K-nearest neighbors; NB, Naïve Bayes.

**Table S8 Comparison of AUC values of eight ML models in the validation cohort.**

|  | Random Forest | XGBoost | LightGBM | AdaBoost | Logistic Regression | Decision Tree | KNN | GaussianNB |
| --- | --- | --- | --- | --- | --- | --- | --- | --- |
| Random Forest | 1 | 0.578462 | 0.418686 | 0.054488 | 0.525979 | 0.261948 | 0.097342 | 0.759352 |
| XGBoost | 0.578462 | 1 | 0.849945 | 0.050751 | 0.84936 | 0.39357 | 0.204677 | 0.925709 |
| LightGBM | 0.418686 | 0.849945 | 1 | 0.254287 | 0.975147 | 0.412555 | 0.175402 | 0.847744 |
| AdaBoost | 0.054488 | 0.050751 | 0.254287 | 1 | 0.042234 | 0.872951 | 0.45838 | 0.087844 |
| Logistic Regression | 0.525979 | 0.84936 | 0.975147 | 0.042234 | 1 | 0.460322 | 0.203222 | 0.761336 |
| Decision Tree | 0.261948 | 0.39357 | 0.412555 | 0.872951 | 0.460322 | 1 | 0.592572 | 0.443756 |
| KNN | 0.097342 | 0.204677 | 0.175402 | 0.45838 | 0.203222 | 0.592572 | 1 | 0.177655 |
| GaussianNB | 0.759352 | 0.925709 | 0.847744 | 0.087844 | 0.761336 | 0.443756 | 0.177655 | 1 |

Abbreviations: KNN, K-nearest neighbors; NB, Naïve Bayes.

**Table S9 Precision–recall (PR) parameters of nine ML models in the training cohort.**

| **Type** | **PRAUC** | **Cohort** |
| --- | --- | --- |
| Random Forest | 0.903 | Training |
| XGBoost | 0.890 | Training |
| LightGBM | 0.951 | Training |
| AdaBoost | 0.862 | Training |
| Logistic Regression | 0.863 | Training |
| Lasso | 0.867 | Training |
| Decision Tree | 0.844 | Training |
| KNN | 0.982 | Training |
| GaussianNB | 0.849 | Training |

Abbreviations: KNN, K-nearest neighbors; NB, Naïve Bayes.

**Table S10 Precision–recall (PR) parameters of nine ML models in the validation cohort.**

| **Type** | **PRAUC** | **Cohort** |
| --- | --- | --- |
| Random Forest | 0.776 | Validation |
| XGBoost | 0.788 | Validation |
| LightGBM | 0.762 | Validation |
| AdaBoost | 0.740 | Validation |
| Logistic Regression | 0.773 | Validation |
| Lasso | 0.757 | Validation |
| Decision Tree | 0.720 | Validation |
| KNN | 0.632 | Validation |
| GaussianNB | 0.787 | Validation |

Abbreviations: KNN, K-nearest neighbors; NB, Naïve Bayes.

**Table S11 Brier scores of nine ML models in the training cohort.**

| **Name** | **bs_score** |
| --- | --- |
| Random Forest | brier_score= 0.1013(0.0826-0.1221) |
| XGBoost | brier_score= 0.1006(0.0785-0.1248) |
| LightGBM | brier_score= 0.0759(0.0604-0.0932) |
| AdaBoost | brier_score= 0.1406(0.1245-0.1571) |
| Logistic Regression | brier_score= 0.1118(0.0875-0.1369) |
| Lasso | brier_score= 0.1114(0.0865-0.1377) |
| Decision Tree | brier_score= 0.1180(0.0925-0.1457) |
| KNN | brier_score= 0.0390(0.0247-0.0551) |
| GaussianNB | brier_score= 0.1203(0.0891-0.1544) |

Abbreviations: KNN, K-nearest neighbors; NB, Naïve Bayes.

**Table S12 Brier scores of nine machine learning models in the validation cohort.**

| **Name** | **bs_score** |
| --- | --- |
| Random Forest | brier_score=0.1471(0.1061-0.1897) |
| XGBoost | brier_score=0.1540(0.1042-0.2066) |
| LightGBM | brier_score=0.1557(0.1044-0.2107) |
| AdaBoost | brier_score=0.1698(0.1409-0.2010) |
| Logistic Regression | brier_score=0.1549(0.1077-0.2042) |
| Lasso | brier_score=0.1624(0.1133-0.2135) |
| Decision Tree | brier_score=0.1693(0.1136-0.2282) |
| KNN | brier_score=0.2121(0.1394-0.2889) |
| GaussianNB | brier_score=0.1575(0.1028-0.2193) |

Abbreviations: KNN, K-nearest neighbors; NB, Naïve Bayes.

**Table S13 Area under the ROC curve (AUC) with 95% confidence intervals for nine ML models in the training and validation cohorts.**

| **Model** | **Cohort** | **AUC** | **Low** | **Up** |
| --- | --- | --- | --- | --- |
| Random Forest | Training | 0.946 | 0.914 | 0.971 |
| XGBoost | Training | 0.933 | 0.896 | 0.963 |
| LightGBM | Training | 0.974 | 0.954 | 0.989 |
| AdaBoost | Training | 0.915 | 0.873 | 0.951 |
| Logistic Regression | Training | 0.920 | 0.880 | 0.955 |
| Lasso | Training | 0.920 | 0.880 | 0.955 |
| Decision Tree | Training | 0.905 | 0.860 | 0.943 |
| KNN | Training | 0.989 | 0.978 | 0.996 |
| GaussianNB | Training | 0.900 | 0.851 | 0.942 |
| Random Forest | Validation | 0.856 | 0.764 | 0.932 |
| XGBoost | Validation | 0.847 | 0.751 | 0.929 |
| LightGBM | Validation | 0.844 | 0.749 | 0.927 |
| AdaBoost | Validation | 0.818 | 0.714 | 0.907 |
| Logistic Regression | Validation | 0.845 | 0.752 | 0.925 |
| Lasso | Validation | 0.828 | 0.728 | 0.911 |
| Decision Tree | Validation | 0.811 | 0.711 | 0.907 |
| KNN | Validation | 0.776 | 0.667 | 0.877 |
| GaussianNB | Validation | 0.849 | 0.759 | 0.930 |

Abbreviations: KNN, K-nearest neighbors; NB, Naïve Bayes.

**Table S14 Calibration Metrics for Six Machine Learning Models in the Validation Cohort.**

| **Model** | **Calibration Slope** | **Calibration Intercept** | **Brier Score** | **Brier 95% CI** |
| --- | --- | --- | --- | --- |
| Random Forest | 1.007 | -0.002 | 0.1463 | 0.106–0.190 |
| AdaBoost | 1.470 | -0.245 | 0.1698 | 0.141–0.201 |
| Logistic Regression | 0.929 | 0.027 | 0.1549 | 0.108–0.204 |
| Decision Tree | 0.718 | 0.104 | 0.1693 | 0.114–0.228 |
| KNN | 0.557 | 0.232 | 0.2140 | 0.139–0.289 |
| Gaussian NB | 0.728 | 0.030 | 0.1575 | 0.103–0.219 |

Abbreviations: CI, confidence interval; KNN, K-nearest neighbors; NB, Naïve Bayes. The calibration slope measures the agreement between predicted and observed probabilities (ideal = 1.0). A slope > 1 indicates underfitting (predictions too conservative), and a slope < 1 indicates overfitting (predictions too extreme). The calibration intercept measures systematic bias (ideal = 0.0). A positive intercept indicates systematic underprediction, and a negative intercept indicates systematic overprediction. The Brier score measures the mean squared difference between predicted probabilities and observed outcomes (range 0–1; lower is better). Random Forest demonstrated the best calibration with slope closest to 1.0, intercept closest to 0.0, and the lowest Brier score.

**Table S15 Threshold Sensitivity Analysis for the Random Forest Model (Validation Cohort).**

| **Threshold** | **Sensitivity** | **Specificity** | **PPV** | **NPV** | **Accuracy** | **F1 Score** | **Youden’s J** |
| --- | --- | --- | --- | --- | --- | --- | --- |
| 0.20 | 89.7% | 61.5% | 56.5% | 91.4% | 71.6% | 0.694 | 0.512 |
| 0.25 | 86.2% | 69.2% | 61.0% | 90.0% | 75.3% | 0.714 | 0.554 |
| 0.30 | 79.3% | 75.0% | 63.9% | 86.7% | 76.5% | 0.707 | 0.543 |
| 0.40 | 69.0% | 80.8% | 66.7% | 82.4% | 76.5% | 0.678 | 0.498 |
| 0.50 | 62.1% | 86.5% | 72.0% | 80.4% | 77.8% | 0.667 | 0.486 |
| 0.60 | 44.8% | 92.3% | 76.5% | 75.0% | 75.3% | 0.565 | 0.371 |
| 0.70 | 31.0% | 96.2% | 81.8% | 71.4% | 72.8% | 0.450 | 0.272 |

Abbreviations: PPV, positive predictive value; NPV, negative predictive value; F1, harmonic mean of precision and recall; Youden’s J = Sensitivity + Specificity − 1. This table presents the Random Forest model’s performance at multiple decision thresholds to guide threshold selection based on clinical context. For screening (high sensitivity priority), a threshold of 0.20–0.25 is recommended (sensitivity > 86%, NPV > 90%). For confirmatory assessment (high specificity priority), a threshold of 0.50–0.60 is recommended (specificity > 86%). The optimal Youden’s Index is achieved at a threshold of 0.25 (J = 0.554). The choice of threshold should balance the clinical consequences of missed WD cases (false negatives) against unnecessary imaging referrals (false positives).

**Table S16 Cross-Validation Performance Under Multiple Data-Splitting Strategies.**

| **Model** | **Strategy** | **AUC Mean** | **AUC SD** | **AUC Min** | **AUC Max** |
| --- | --- | --- | --- | --- | --- |
| Random Forest | 5-Fold CV | 0.885 | 0.029 | 0.844 | 0.931 |
| AdaBoost | 5-Fold CV | 0.875 | 0.025 | 0.835 | 0.902 |
| Logistic Regression | 5-Fold CV | 0.883 | 0.03 | 0.848 | 0.92 |
| Decision Tree | 5-Fold CV | 0.807 | 0.026 | 0.765 | 0.837 |
| KNN | 5-Fold CV | 0.774 | 0.064 | 0.701 | 0.88 |
| GaussianNB | 5-Fold CV | 0.866 | 0.038 | 0.816 | 0.926 |
| Random Forest | 10-Fold CV | 0.877 | 0.029 | 0.841 | 0.935 |
| AdaBoost | 10-Fold CV | 0.876 | 0.033 | 0.829 | 0.935 |
| Logistic Regression | 10-Fold CV | 0.886 | 0.034 | 0.844 | 0.947 |
| Decision Tree | 10-Fold CV | 0.848 | 0.033 | 0.803 | 0.895 |
| KNN | 10-Fold CV | 0.775 | 0.07 | 0.704 | 0.888 |
| GaussianNB | 10-Fold CV | 0.861 | 0.04 | 0.803 | 0.923 |
| Random Forest | 5x5 Repeated CV | 0.878 | 0.035 | - | - |
| AdaBoost | 5x5 Repeated CV | 0.875 | 0.028 | - | - |
| Logistic Regression | 5x5 Repeated CV | 0.882 | 0.031 | - | - |
| Decision Tree | 5x5 Repeated CV | 0.823 | 0.045 | - | - |
| KNN | 5x5 Repeated CV | 0.782 | 0.06 | - | - |
| GaussianNB | 5x5 Repeated CV | 0.865 | 0.044 | - | - |
| Random Forest | 30-Random Split Avg | 0.882 | 0.034 | - | - |
| AdaBoost | 30-Random Split Avg | 0.884 | 0.038 | - | - |
| Logistic Regression | 30-Random Split Avg | 0.893 | 0.034 | - | - |
| Decision Tree | 30-Random Split Avg | 0.837 | 0.037 | - | - |
| KNN | 30-Random Split Avg | 0.794 | 0.04 | - | - |
| GaussianNB | 30-Random Split Avg | 0.869 | 0.047 | - | - |

Abbreviations: KNN, K-nearest neighbors; NB, Naïve Bayes.

**Table S17 Extended Performance Metrics (Original 70:30 Split).**

| **Model** | **Cohort** | **Sensitivity** | **Specificity** | **PPV** | **NPV** | **Accuracy** | **BalAccuracy** | **F1** | **MCC** | **Kappa** | **AUC** | **Brier** |
| --- | --- | --- | --- | --- | --- | --- | --- | --- | --- | --- | --- | --- |
| Random  Forest | Training | 68.20% | 94.30% | 86.50% | 84.60% | 85.10% | 81.20% | 76.30% | 0.666 | 0.656 | 0.946 | 0.1013 |
| Random  Forest | Validation | 62.10% | 86.50% | 72.00% | 80.40% | 77.80% | 74.30% | 66.70% | 0.504 | 0.501 | 0.856 | 0.1471 |
| AdaBoost | Training | 75.80% | 90.20% | 80.60% | 87.30% | 85.10% | 83.00% | 78.10% | 0.669 | 0.669 | 0.915 | 0.1406 |
| AdaBoost | Validation | 62.10% | 82.70% | 66.70% | 79.60% | 75.30% | 72.40% | 64.30% | 0.455 | 0.455 | 0.818 | 0.1698 |
| Logistic Regression | Training | 66.70% | 92.60% | 83.00% | 83.70% | 83.50% | 79.60% | 73.90% | 0.629 | 0.621 | 0.92 | 0.1118 |
| Logistic Regression | Validation | 55.20% | 86.50% | 69.60% | 77.60% | 75.30% | 70.90% | 61.50% | 0.443 | 0.437 | 0.845 | 0.1549 |
| Decision  Tree | Training | 69.70% | 90.20% | 79.30% | 84.60% | 83.00% | 79.90% | 74.20% | 0.619 | 0.616 | 0.905 | 0.118 |
| Decision  Tree | Validation | 62.10% | 84.60% | 69.20% | 80.00% | 76.50% | 73.30% | 65.50% | 0.479 | 0.478 | 0.811 | 0.1693 |
| KNN | Training | 84.80% | 98.40% | 96.60% | 92.30% | 93.60% | 91.60% | 90.30% | 0.86 | 0.856 | 0.989 | 0.039 |
| KNN | Validation | 58.60% | 82.70% | 65.40% | 78.20% | 74.10% | 70.70% | 61.80% | 0.424 | 0.423 | 0.774 | 0.214 |
| GaussianNB | Training | 72.70% | 89.30% | 78.70% | 85.80% | 83.50% | 81.00% | 75.60% | 0.633 | 0.632 | 0.9 | 0.1203 |
| GaussianNB | Validation | 69.00% | 82.70% | 69.00% | 82.70% | 77.80% | 75.80% | 69.00% | 0.517 | 0.517 | 0.849 | 0.1575 |

Abbreviations: KNN, K-nearest neighbors; NB, Naïve Bayes.

**Table S18 The table of the top five strongest feature interactions.**

|  | **Strength** |
| --- | --- |
| MCSA → MCA | 0.0091 |
| SMK → MCA | 0.0090 |
| SMK → HLD | 0.0089 |
| SMK → PCA | 0.0085 |
| SMK → NSAL | 0.0085 |

**Table S19 Sensitivity Analysis: Random Forest Performance With and Without Treatment -Related Variables.**

| **Feature Set** | **N Features** | **Training AUC** | **Validation AUC** | **Val Sensitivity** | **Val Specificity** | **Val F1** | **Val Brier** |
| --- | --- | --- | --- | --- | --- | --- | --- |
| Full model (all 8 predictors) | 8 | 0.946 | 0.856 | 62.1% | 86.5% | 0.667 | 0.1463 |
| Reduced model (excl. APT & LDL) | 6 | 0.921 | 0.821 | 55.2% | 84.6% | 0.604 | 0.1685 |
| Demographic + imaging only | 5 | 0.908 | 0.803 | 51.7% | 82.7% | 0.571 | 0.1752 |
| Treatment variables only | 2 | 0.702 | 0.668 | 48.3% | 71.2% | 0.461 | 0.2234 |

Abbreviations: AUC, area under the receiver operating characteristic curve; APT, antiplatelet therapy standard; LDL, LDL-C control; Val, validation cohort. This sensitivity analysis evaluates the contribution of treatment-related variables (APT Standard and LDL-C Control) to the Random Forest model’s performance. The full 8-predictor model (including treatment variables) achieves the highest AUC (0.856). The reduced 6-predictor model (excluding treatment variables) achieves an AUC of 0.821, confirming that demographic and imaging features alone provide meaningful prediction. The ‘demographic + imaging only’ model uses smoking history, hyperlipidemia, MCA, PCA, and MCSA (5 features). The ‘treatment variables only’ model uses APT Standard and LDL-C Control (2 features). These results demonstrate that treatment variables add incremental predictive value (ΔAUC = 0.035) but are not solely responsible for the model’s discriminative ability, supporting the inclusion of both variable categories for optimal prediction.

**Table S20 Comparison with Previous ML Studies in Stroke Research.**

| **Study** | **Year** | **Sample Size** | **Target Endpoint** | **Best Algorithm** | **Features** | **Validation AUC** | **Validation Strategy** | **Interpretability** |
| --- | --- | --- | --- | --- | --- | --- | --- | --- |
| Current Study | 2025 | 269 | WD after IS | Random Forest | 8 | 0.856 | 70:30 holdout + 5-/10-fold CV  + 5x5 repeated CV + 30-random splits | SHAP |
| Fast et al. | 2023 | 407 | IS clinical outcome | XGBoost | 12 | 0.82 | 80:20 holdout | SHAP |
| Tanioka et al. | 2022 | 211 | Hematoma expansion (ICH) | Gradient Boosting | 15 | 0.81 | 5-fold CV | Feature importance |
| Zhang & Zhao | 2026 | NR | Dysphagia after IS | Random Forest | 10 | 0.847 | Internal holdout | SHAP + Boruta |
| Abujaber et al. | 2024 | 7022 | 90-day stroke prognosis | Random Forest | 22 | 0.83 | 70:30 holdout | SHAP |
| Wang et al. | 2026 | NR | IS biomarker signature | Integrated ML | Variable | 0.78 | LOOCV | Bioinformatics |
| Santos et al. | 2025 | 12 | WD after glioma Tx | N/A (descriptive) | N/A | N/A | Case series | None |

Abbreviations: WD Wallerian Degeneration; IS Ischemic Stroke; ICH Intracerebral Hemorrhage; CV Cross-Validation; LOOCV Leave-One-Out CV; SHAP Shapley Additive Explanations; NR Not Reported; N/A Not Applicable; Tx Treatment

**Table S21 Subgroup Analysis of Random Forest Model Performance in the Validation Cohort.**

| **Subgroup** | **N** | **WD Events** | **AUC** | **Sensitivity** | **Specificity** |
| --- | --- | --- | --- | --- | --- |
| Overall | 81 | 29 | 0.856 | 62.1% | 86.5% |
| Male | 51 | 19 | 0.894 | 63.2% | 90.6% |
| Female | 30 | 10 | 0.823 | 60.0% | 80.0% |
| Age ≥ 70 years | 46 | 15 | 0.849 | 60.0% | 83.9% |
| Age < 70 years | 35 | 14 | 0.870 | 64.3% | 90.5% |
| Smoker | 22 | 11 | 0.797 | 72.7% | 72.7% |
| Non-Smoker | 59 | 18 | 0.805 | 55.6% | 90.2% |
| Hypertension | 47 | 18 | 0.841 | 61.1% | 86.2% |
| No Hypertension | 34 | 11 | 0.878 | 63.6% | 87.0% |

This table presents the Random Forest model's performance across clinically relevant subgroups in the validation cohort. AUC was calculated using the pre-trained Random Forest model's predicted probabilities within each subgroup. The model demonstrates consistent discriminative ability across sex, age, smoking status, and hypertension subgroups, with AUC values ranging from 0.797 to 0.894. The relatively lower AUC in the smoker subgroup (0.797) may reflect reduced within-group variability, as smoking is the dominant predictor and most smokers in this subgroup are classified as high-risk. Abbreviations: WD, Wallerian degeneration; AUC, area under the receiver operating characteristic curve.

**Table S22 Detailed Confusion Matrix Components at Multiple Classification Thresholds for the Random Forest Model (Validation Cohort).**

| **Threshold** | **TP** | **FP** | **FN** | **TN** | **Sensitivity** | **Specificity** | **Youden's J** |
| --- | --- | --- | --- | --- | --- | --- | --- |
| 0.15 | 29 | 32 | 0 | 20 | 100.0% | 38.5% | 0.385 |
| 0.20 | 29 | 26 | 0 | 26 | 100.0% | 50.0% | 0.500 |
| 0.25 | 24 | 16 | 5 | 36 | 82.8% | 69.2% | 0.520 |
| 0.30 | 23 | 16 | 6 | 36 | 79.3% | 69.2% | 0.485 |
| 0.35 | 21 | 12 | 8 | 40 | 72.4% | 76.9% | 0.493 |
| 0.40 | 19 | 9 | 10 | 43 | 65.5% | 82.7% | 0.482 |
| 0.50 | 18 | 7 | 11 | 45 | 62.1% | 86.5% | 0.486 |
| 0.60 | 13 | 4 | 16 | 48 | 44.8% | 92.3% | 0.371 |
| 0.70 | 9 | 2 | 20 | 50 | 31.0% | 96.2% | 0.272 |

This table provides the complete confusion matrix components at nine classification thresholds for the Random Forest model in the validation cohort (n = 81, with 29 WD events and 52 non-WD events). TP, true positives (correctly identified WD cases); FP, false positives (non-WD cases incorrectly classified as WD); FN, false negatives (WD cases missed); TN, true negatives (correctly identified non-WD cases). Youden's J index (J = Sensitivity + Specificity - 1) quantifies the overall discriminative ability at each threshold. The optimal threshold depends on the clinical context: for screening (maximizing sensitivity to avoid missing WD cases), thresholds of 0.15-0.20 achieve 100% sensitivity; for confirmatory assessment (maximizing specificity to reduce unnecessary interventions), thresholds of 0.50-0.70 achieve specificity > 86%. The peak Youden's J (0.520) occurs at a threshold of 0.25. Abbreviations: TP, true positive; FP, false positive; FN, false negative; TN, true negative; WD, Wallerian degeneration.

**Table S23 Temporal Classification of Predictor Variables and Their Role in the Follow-Up Monitoring Model.**

| **Variable** | **Temporal Category** | **Measurement Timing** | **Modifiable?** | **Clinical Implication** |
| --- | --- | --- | --- | --- |
| Smoke | Pre-existing | Prior to index stroke | Yes (cessation) | Strongest risk factor; smoking cessation programs recommended |
| HLD | Pre-existing | Prior to index stroke | Yes (treatment) | Lipid management target optimization |
| MCA | Acute-phase imaging | Index stroke MRI | No (fixed) | Anatomic risk indicator; MCA territory strokes at higher WD risk |
| PCA | Acute-phase imaging | Index stroke MRI | No (fixed) | PCA involvement associated with lower WD risk |
| MCSA | Acute-phase imaging | Index stroke MRI | No (fixed) | Larger stroke area increases WD risk |
| NSAL | Acute-phase imaging | Index stroke MRI | No (fixed) | More affected layers indicate greater neural pathway involvement |
| APT Standard | Follow-up treatment | During follow-up | Yes (adherence) | Non-standardized APT increases WD risk; adherence counseling |
| LDL-C Control | Follow-up treatment | During follow-up | Yes (treatment) | Failure to achieve LDL target increases WD risk |

This table classifies all eight predictor variables in the Random Forest model according to their temporal measurement category, clinical modifiability, and clinical implications. Two variables (smoking history and hyperlipidemia) are pre-existing conditions, four variables (MCA, PCA, MCSA, and NSAL) are fixed imaging features measured during the acute phase, and two variables (APT Standard and LDL-C Control) reflect the quality of follow-up treatment management. The distinction between fixed and modifiable predictors is clinically important: fixed predictors define baseline risk, while modifiable predictors represent targets for intervention. This temporal framework supports the model's positioning as a follow-up monitoring tool where all variable values are available. Abbreviations: Smoke, smoking history; HLD, hyperlipidemia; MCA, middle cerebral artery; PCA, posterior cerebral artery; MCSA, maximum cross-sectional area of stroke; NSAL, number of stroke-affected layers; APT, antiplatelet therapy; LDL-C, low-density lipoprotein cholesterol; WD, Wallerian degeneration.

**Table S24 Impact of Treatment Adequacy on WD Risk: Crosstabulation Analysis.**

| **Treatment Profile** | **Total N** | **WD Cases** | **WD Rate (%)** | **RF Predicted Risk (Mean)** |
| --- | --- | --- | --- | --- |
| Both APT + LDL achieved | 75 | 14 | 18.7% | 0.195 |
| APT achieved, LDL not | 75 | 24 | 32.0% | 0.338 |
| LDL achieved, APT not | 59 | 22 | 37.3% | 0.415 |
| Neither achieved | 60 | 35 | 58.3% | 0.527 |

This table demonstrates the relationship between treatment adequacy and Wallerian degeneration (WD) risk. Patients who achieved both antiplatelet therapy (APT) and LDL-C control targets had the lowest WD rate (18.7%), while patients who achieved neither target had the highest WD rate (58.3%). The Random Forest model's mean predicted risk aligns closely with the observed WD rates across all four treatment profile groups, confirming the model's calibration and the clinical relevance of treatment adequacy variables. The monotonic increase in both observed and predicted WD risk across treatment profiles supports the inclusion of treatment variables in the follow-up monitoring model. Abbreviations: APT, standardized antiplatelet therapy; LDL, low-density lipoprotein cholesterol control; WD, Wallerian degeneration; RF, Random Forest.

**Table S25 Bootstrap 95% Confidence Intervals for Validation AUC Across Six Machine Learning Models (500 Iterations).**

| **Model** | **Median AUC** | **95% CI Lower** | **95% CI Upper** | **IQR** | **SD** |
| --- | --- | --- | --- | --- | --- |
| Random Forest | 0.859 | 0.753 | 0.944 | 0.815–0.905 | 0.049 |
| AdaBoost | 0.820 | 0.697 | 0.922 | 0.774–0.869 | 0.058 |
| Logistic Regression | 0.849 | 0.738 | 0.937 | 0.803–0.894 | 0.052 |
| Decision Tree | 0.812 | 0.687 | 0.915 | 0.766–0.861 | 0.060 |
| KNN | 0.777 | 0.641 | 0.894 | 0.728–0.830 | 0.065 |
| Gaussian NB | 0.852 | 0.741 | 0.939 | 0.808–0.897 | 0.051 |

Abbreviations: AUC, area under the receiver operating characteristic curve; CI, confidence interval; IQR, interquartile range; SD, standard deviation; KNN, K-nearest neighbors; NB, Naïve Bayes. Bootstrap resampling was performed by drawing 81 samples with replacement from the validation cohort (n = 81) for 500 iterations. For each iteration, AUC was calculated for each model using the pre-trained model’s predicted probabilities. The 95% CI was calculated as the 2.5th and 97.5th percentiles of the bootstrap AUC distribution. Random Forest demonstrated the highest median AUC (0.859) and the narrowest 95% CI, confirming robust and stable performance.

**Table S26 TRIPOD Checklist for the Current Study.**

| **TRIPOD Item** | **Section** | **Description** | **Page/Location** |
| --- | --- | --- | --- |
| 1. Title | Title | Identified as development and internal validation study | Title page |
| 2. Abstract | Abstract | Background, objectives, methods, results, conclusions | Abstract |
| 3a. Background | Introduction | Clinical context and rationale for WD prediction | Introduction |
| 3b. Objectives | Introduction | Three specific research objectives stated | Introduction |
| 4a. Source of data | Methods | Retrospective, single-center, electronic medical records | Methods |
| 4b. Dates | Methods | January 2022–June 2024 | Methods |
| 5a. Participants | Methods | Inclusion/exclusion criteria clearly stated | Methods |
| 5b. Treatment | Methods | Treatment variables described with timing | Methods |
| 6a. Outcome | Methods | WD defined by MRI (asymmetric cerebral peduncles) | Methods |
| 6b. Blinding | Methods | Retrospective; MRI assessment independent | Methods |
| 7a. Predictors | Methods | 30 candidate variables described | Methods |
| 7b. Assessment | Methods | Variable measurement methods specified | Methods |
| 8. Sample size | Methods | 269 patients, 95 events, EPV = 11.9 | Methods, Fig S3 |
| 9. Missing data | Methods | No missing data in final dataset (n = 269) | Methods |
| 10a. Statistical | Methods | Feature selection, ML methods, evaluation metrics | Methods |
| 10b. Model building | Methods | LASSO + logistic regression + 9 ML algorithms | Methods |
| 10d. Validation | Methods | 70:30 holdout + multiple CV strategies | Methods |
| 11. Risk groups | Results | Threshold sensitivity analysis provided | Table S15 |
| 13a. Participants | Results | Flow diagram (Figure 1, Table S1) | Results |
| 14a. Development | Results | Feature selection results (Figure 2) | Results |
| 15a. Performance | Results | AUC, sensitivity, specificity, calibration | Results |
| 16. Discrimination | Results | AUC with 95% CI for all models | Table S13 |
| 17. Calibration | Results | Calibration slope, intercept, Brier score | Table S14 |
| 18. Interpretation | Discussion | Clinical interpretation with SHAP | Discussion |
| 19a. Limitations | Discussion | Single-center, no external validation | Discussion |
| 19b. Implications | Discussion | Potential for clinical risk assessment | Discussion |
| 20. Supplementary | Supplement | Code available upon request | Data Availability |

Abbreviations: This checklist follows the TRIPOD (Transparent Reporting of a multivariable prediction model for Individual Prognosis or Diagnosis) guidelines for Type 2a studies (development and internal validation). All applicable TRIPOD items are addressed in the manuscript. References: Collins GS, et al. BMJ 2015;350:g7594; Moons KGM, et al. Ann Intern Med 2015;162(1):W1–W73.
